# Effects of common antibiotics on representative gut bacterial strains associated with inflammatory bowel disease

**DOI:** 10.1186/s12866-026-05459-w

**Published:** 2026-08-06

**Authors:** Pallabita Saha, Lucas Böttcher, Didier Gonze, Xingjian Zhou, Kristel Bernaerts, Karoline Faust

**Affiliations:** 1 Department of Microbiology, Immunology and Transplantation, Rega Institute for Medical Research, Laboratory of Molecular Bacteriology, KU Leuven, Leuven, Belgium; 2 Department of Computational Science and Philosophy, Frankfurt School of Finance and Management, Frankfurt a. M, Germany; 3 Unité de Chronobiologie Théorique, Faculté des Sciences, Université Libre de Bruxelles, Bruxelles, Belgium; 4 Department of Chemical Engineering, Chemical and Biochemical Reactor Engineering and Safety (CREaS), KU Leuven, Leuven, Belgium

**Keywords:** Antibiotics, Inflammatory bowel disease, Gut microbiome, Growth dynamics

## Abstract

**Background:**

Inflammatory Bowel Disease (IBD), a group of multifactorial diseases marked by chronic inflammation of the gastrointestinal tract, is characterized by an altered gut microbial community composition that is enriched in opportunistic and pathogenic species, such as *Bacteroides fragilis* and adherent-invasive *Escherichia coli* strains. To better understand the consequences of using antibiotics to suppress these harmful species, we quantified the response of three bacterial species representative of a healthy gut microbiome, as well as two species typically enriched in IBD to five selected antibiotics that are widely employed against anaerobes.

**Results:**

We found that the adherent-invasive *Escherichia coli* LF82 strain and the type strains of the opportunistic Bacteroides species *B. fragilis* and *B. thetaiotaomicron* are resistant to even high doses of several of the tested antibiotics whereas the type strains of the probiotic butyrate producer *Roseburia intestinalis* and the acetogen *Blautia hydrogenotrophica* are sensitive to the majority of the tested antibiotics at low concentrations. We performed a Bayesian parameter estimation of a parsimonious mechanistic growth model. The effects of the antibiotics on lag phase, growth rate, and death rate varied across compounds in a manner consistent with their modes of action. For example, metronidazole, which inhibits DNA synthesis, reduced the growth rate, whereas meropenem, which inhibits cell wall synthesis, increased the death rate in several cases. In contrast, eravacycline, piperacillin/tazobactam and rifampin exhibited variable and species-dependent effects on the growth dynamics.

**Conclusion:**

The observation that opportunistic strains in monoculture are resistant to several tested antibiotics in contrast to commensals questions the use of broad-spectrum antibiotics for microbiome modulation in gastrointestinal diseases and motivates the need for targeted strategies to reshape dysbiotic microbial communities.

**Supplementary Information:**

The online version contains supplementary material available at https://doi.org/10.1186/s12866-026-05459-w.

## Background

The human colon (gut) microbiome is the most dense and diverse human-associated microbiome and contributes to host health by degrading fibers, synthesizing essential vitamins, and protecting the host from pathogens [1, 2]. Three variations of gut microbiota, referred to as enterotypes, have been described for healthy adults [3, 4], which are dominated by Ruminococcus, Bacteroides and Prevotella species, respectively. Inflammatory Bowel Disease (IBD) is a multifactorial group of diseases characterized by chronic inflammation of the gastrointestinal (GI) tract. Its main types are Crohn’s Disease (CD), where the entire GI tract can be affected, and Ulcerative Colitis, which is restricted to the colon and rectum. IBD features a “dysbiotic” gut microbiome that significantly differs from that of healthy controls [5, 6] and is deemed less healthy due to the depletion of probiotic commensals and the enrichment of opportunistic and even pathogenic species. In CD, the relative abundance of butyrate producers such as *Faecalibacterium duncaniae* and *Roseburia* species is typically reduced, whereas fast-growing opportunistic species such as *Bacteroides fragilis* and *Escherichia coli* are enriched compared to healthy gut microbiome variants [7–11]. Butyrate, which is consumed by epithelial cells, generally has a positive effect on gut health [12] and butyrate producers such as *Faecalibacterium duncaniae* and *Roseburia intestinalis* are therefore regarded as probiotic candidate species [13, 14].

To date, it is unclear whether the shift in gut microbial composition observed in IBD is a consequence or a cause of the impaired epithelial barrier, which allows microorganisms to reach intestinal tissue [15]. However, several observations point to the latter interpretation, including fecal transfer experiments in mouse models, the observation that ileal lesions heal when shielded from intestinal luminal material and the partial success of fecal microbiome transplants as a CD treatment [16–18]. Since IBD is characterized by an enrichment in pathogens, such as the adherent invasive *E. coli* (AIEC) strain LF82 that can enter epithelial cells [19], it is desirable to return the gut microbiota to a healthier state.

In this context, we investigated the impact of antibiotics belonging to five different classes targeting anaerobic gut bacterial species representing the healthy and CD gut microbiome. We selected antibiotics that are either directly employed (metronidazole [20–22]) or that represent a class of antibiotics commonly used (rifampin of the rifamycin class) [23] in IBD treatment. We also included antibiotics typically applied in case of complications such as intra-abdominal infections (piperacillin combined with tazobactam [24, 25]), or sepsis (meropenem [26]). Finally, we added the next-generation antibiotic eravacycline, a fluorocycline that was developed to overcome classical tetracycline resistance mechanisms and that has been evaluated in the context of intra-abdominal infections but has not yet been systematically studied for IBD-associated gut bacteria [27, 28]. Metronidazole, the most extensively studied antibiotic for CD [20, 29], has been shown to induce significant shifts in the gut microbial composition, which included reduction in obligate anaerobes and butyrate-producing taxa [22, 30]. However, the effects of IBD-relevant antibiotics on the growth of gut bacterial species are still underexplored.

AIEC strains, including LF82, have been reported to persist under antibiotic exposure in experimental studies [31, 32]. AIEC strains are capable of surviving β-lactam antibiotics treatment and exhibit enhanced tolerance compared to non-AIEC strains, particularly under conditions that are similar to the intestinal environment [33]. This persistence has been attributed to a combination of intrinsic resistance mechanisms, efflux pump activity [34] and the ability to adapt to physiological stressors. This has raised concerns regarding the failure of antibiotic treatment to eradicate AIEC while simultaneously exacerbating dysbiosis [35].

Members of the *Bacteroides fragilis* group have been the focus of numerous susceptibility studies because of their abundance in the human gut and their frequent exposure to antibiotics [36, 37]. While *B. fragilis* has previously been considered susceptible to metronidazole and carbapenems, resistance to multiple antibiotic classes - including β-lactams - has been increasingly reported, especially in clinical isolates [38–41]. On the other hand, *Bacteroides thetaiotaomicron* displays distinct strain-dependent variability in antibiotic susceptibility [37, 42, 43]. These findings emphasize the difficulty of predicting antibiotic responses at the species level within the genus *Bacteroides*.

Prior clinical and experimental studies indicate that the antibiotics commonly administered in IBD treatment exert a strong effect on the gut microbial communities [21, 22, 44, 45] but since strain-level resolution is rarely achieved [46, 47], it is not clear whether antibiotic treatment specifically suppresses IBD-associated pathobionts such as AIEC. This motivates a systematic and quantitative comparison of antibiotic effects on representative commensal and IBD-associated gut bacteria under controlled conditions. Here, we selected the type strains of the butyrate producer *R. intestinalis*, the acetogen *Blautia hydrogenotrophica* and the primary fermenter *Bacteroides thetaiotaomicron* as representative commensals, which are common members of the healthy, Bacteroides-type gut microbiome [4]. In addition, we chose the type strain of *B. fragilis* and *E. coli* LF82 as representative strains of a dysbiotic CD community. We then quantified the impact of the five selected antibiotics, added in different doses, on the growth of these five species in vitro (see Additional file 1: Supplementary Figure S1).

## Methods

### Bacterial strains and culture conditions

The following five human gut bacterial strains were used: *Bacteroides thetaiotaomicron* VPI 5482, *Blautia hydrogenotrophica* DSM 10507, *Roseburia intestinalis* L1-82, *Escherichia coli* LF82 and *Bacteroides fragilis* NCTC 9343. The majority of these are type strains (*Bacteroides thetaiotaomicron* VPI 5482, *Blautia hydrogenotrophica* DSM 10507, *Roseburia intestinalis* L1-82 and *Bacteroides fragilis* NCTC 9343) and were procured from the German Collection of Microorganisms and Cell Cultures GmbH (DSMZ, Germany). *Escherichia coli* LF82 is an AIEC strain and was kindly gifted by Prof. Christel Neut (University of Lille, France). The strains were grown as monocultures inside an anaerobic chamber (Don Whitley HEPA A45 Anaerobic Workstation: 10% H2, 10% CO2, 80% N2, 70% relative humidity) at 37 °C and to prepare the inoculum, 20–50 µL of bacterial stocks (all strains were frozen in Wilkins–Chalgren Anaerobe Broth (WC; Oxoid) plus 20% glycerol) were inoculated in 1000 µL of sterile, deoxygenated WC medium. The precultures were grown for 16–24 h and then transferred to fresh deoxygenated medium for a passage. After 24 h, the cultures were adjusted to an optical density (OD) of ~ 0.1 in WC prior to inoculation. To ensure monoculture purity, verify consistency with the expected culture identity and exclude contamination, 16S rRNA gene Sanger sequencing was performed as an internal quality control measure on precultures before inoculation and on wells after completion of the growth experiments (Additional file 1: Supplementary Table S1). The targeted 16S rRNA gene fragment was amplified with the universal primers fD1 (5′ -AGAGTTTGATCCTGGCTCAG- 3′) and rP2 (5′ -ACGGCTACCTTGTTACGACTT- 3′), yielding an amplicon of approximately 1500 bp. The resulting amplicons were subjected to Sanger sequencing by Eurofins Genomics Sequencing GmbH (Cologne, Germany).

### Antibiotic susceptibility testing using broth dilution method

Antibiotic stock preparation and microdilution plate setup were performed according to the Clinical and Laboratory Standards Institute (CLSI) guidelines (M100) [48], with minor adaptations to media preparation, inoculum standardization, plate assembly and incubation as described below. The antibiotics tested were metronidazole (MP Biomedicals), eravacycline (MedChemExpress), piperacillin (Avantor, Inc.), tazobactam (Avantor, Inc.), meropenem (Avantor, Inc) and rifampin (Avantor, Inc). Stock solutions were prepared in sterile distilled water (filter-sterilized using 0.22 μm syringe filters) or DMSO (diluted with water to keep final DMSO ≤ 2% in any intermediate), depending on solubility, then aliquoted, light-protected and stored at − 20 °C. Two-fold serial dilutions were prepared to generate a concentration range of 0.1, 0.2, 0.4, 0.8 and 1.6 µg/mL. In some experiments, extended concentration ranges of 1, 2, 4, 8 and 16 µg/mL were tested. For piperacillin/tazobactam, concentrations were prepared with tazobactam kept at a fixed concentration of 0.4 µg/mL for the former concentration range and 4 µg/mL for the latter (extended) concentration range. Accordingly, values such as 0.1/0.4, 0.2/0.4, 0.4/0.4, 0.8/0.4 and 1.6/0.4 µg/mL or 1/4, 2/4, 4/4, 8/4 and 16/4 µg/mL, indicate piperacillin concentration together with the fixed tazobactam concentration. On the day of the assay, single-use aliquots were thawed and immediately diluted to working dilutions. Working solutions were prepared fresh inside the anaerobic workstation (Don Whitley A45; 10% H₂, 10% CO₂, 80% N₂) by diluting stocks into sterile, deoxygenated WC medium, thereby minimizing oxygen exposure; no working solutions were stored. β-lactams (meropenem, piperacillin/tazobactam) were prepared fresh for each run to minimize hydrolysis.

Plates were assembled inside the anaerobic workstation. 20 µL of the diluted culture was inoculated into a flat, clear-bottom 96-well plate (Corning 0377) containing 180 µL medium with antibiotics. Wells containing only 200 µL medium served as blanks (negative controls) and wells with inoculum but no antibiotics served as growth controls (positive controls). For each well, 180 µL of WC (± antibiotic) was dispensed, followed by 20 µL standardized inoculum (final volume 200 µL). Each plate included both positive and negative controls. Each antibiotic concentration was tested in duplicate.

Plates were covered with a lid and placed in the plate reader (VANTAstar, BMG LabTech; plate reader placed inside anaerobic workstation) with a continuous temperature control at 37 °C. Before collecting growth curve data, the experimental set-up was optimized to ensure low measurement noise over extended incubations and high reproducibility across experiments. OD was measured at 600 nm using the matrix scan mode with a diameter of 2 mm and matrix dimension of 4 × 4. This selection was made over single-point, orbital average, and spiral scan modes to minimize well-to-well variability and reduce sensitivity to edge effects. Plates were shaken in double orbital mode at 200 rpm for 5 min prior to each measurement and OD was recorded every 30 min at 37 °C. A volume-based pathlength correction step was added to the protocol to enable direct comparison of OD values measured in the plate reader with the starting OD used for inoculum standardization, which was measured in a 1 cm cuvette using a spectrophotometer. Volume-based correction normalizes OD values to an equivalent 1 cm pathlength based on the liquid present in the well, which improves cross-instrument comparability across experiments. Water-peak–based pathlength correction was avoided, as it is less robust for turbid bacterial cultures where light scattering, medium composition and bubbles can affect absorbance-based pathlength estimation.

### Colony forming unit validation of bactericidal activity of meropenem

To validate that the death rate parameter inferred from OD growth curves corresponds to loss of bacterial viability, colony-forming units (CFUs) were counted for *E. coli* LF82 and *B. fragilis* NCTC 9343 exposed to meropenem — the antibiotic for which OD-based model fitting identified a dose-dependent increase of the death rate in both strains. A 96-well deep-well plate (1 mL volume per well) was prepared with each deep-well containing 900 µL of WC medium supplemented with meropenem at three conditions: no antibiotic (positive control), 0.2 µg/mL (low concentration, sub-MIC), and 2.0 µg/mL (high concentration, supra-MIC). Inoculation was performed by addition of 100 µL of standardized culture at OD600 ~ 0.1, yielding a total volume of 1,000 µL per well. Six independent biological replicates were prepared per condition. Six wells containing only 1000 µL of WC medium served as blanks (negative controls). To assess the reproducibility of the observed growth responses, 200 µL was withdrawn from each well and added to a flat, clear-bottom 96-well plate (Corning 0377) and placed in a plate reader for OD measurements for 26 h using the same protocol as described previously. The deep-well plate containing 800 µL in each well was sealed with an adhesive membrane and incubated statically at 37 °C inside the anaerobic workstation. Samples of 100 µL were withdrawn from the deep well plate at three timepoints: (i) immediately after inoculation (T = 0 h baseline), (ii) at T = 8 h, and (iii) at T = 24 h. Serial 10-fold dilutions were prepared in deoxygenated sterile 0.9% saline inside the anaerobic workstation. Colony enumeration was performed using the drop plate (Miles-Misra [49]) method: 10 µL of each dilution was spotted in five technical replicates per dilution onto Wilkins Chalgren Anaerobe Agar plates pre-equilibrated in the anaerobic workstation for at least 16 h. Plates were incubated anaerobically at 37 °C for 24–48 h. Colonies were enumerated in spots containing 3–30 colonies per 10 µL spot (the countable range for the drop plate method), and CFU/mL was calculated as:

$$\begin{aligned}\mathrm{CFU/mL}&=\text{colony count}/\left(\text{spot volume in mL}\right. \left.\times\text{dilution plated}\right)\end{aligned}$$

with a spot volume of 0.01 mL. A per-replicate mean CFU/mL was computed by averaging across valid spots. Replicates for which all spots fell below the detection limit of the assay (< 300 CFU/mL) are reported as below detection limit. Control wells (no antibiotic) had high CFU counts at all dilutions tested at both T = 8 h and T = 24 h, confirming robust bacterial growth throughout the experiment.

Statistical analysis was performed in R using the lme4 [50] and lmerTest [51] packages. For each species, log₁₀-transformed CFU/mL values were modelled using a linear mixed-effects model with meropenem concentration (control, 0.2 µg/mL, 2.0 µg/mL) and timepoint (T = 8 h, T = 24 h) as fixed effects including their interaction, and biological replicate as a random intercept: log₁₀(CFU/mL) ~ condition × timepoint + (1 | replicate). Individual drop measurements (up to five per biological replicate, with six biological replicates per condition) were used as observations. Zero entries in control wells were treated as missing observations and were excluded from model fitting. Zero entries in meropenem-treated wells were interpreted as genuine below-detection counts and were replaced with a pseudo-count of 1 (log₁₀(1) = 0) to retain these measurements in the analysis. Type III analysis of variance was performed using Satterthwaite’s method. Marginal means were estimated using the emmeans package [52] with orthogonal polynomial contrasts to assess the direction and shape of the dose response at each timepoint, with Kenward-Roger degrees of freedom adjustment.

### Antibiotic resistance gene scanning

Complete genome assemblies for five strains used in this study (*Escherichia coli* LF82, *Bacteroides thetaiotaomicron* VPI-5482, *Bacteroides fragilis* NCTC 9343, *Blautia hydrogenotrophica* DSM 10507, *Roseburia intestinalis* L1-82) were obtained from NCBI using their corresponding assembly accessions. Genome assemblies were analyzed in contig mode using DIAMOND v0.9.25 as the alignment tool [53]. Antimicrobial resistance determinants were identified using RGI v6.0.5 (CARD) [54]. Searches were run locally with an offline installed CARD database (v4.0.1). The hit threshold included only loose hits better than 1 × 10⁻¹⁰, as well as perfect and strict hits.

### Data analysis and statistics

For each plate, background absorbance from blank wells was subtracted from all other wells to obtain corrected OD values. Growth curves (OD versus time) were plotted to assess antibiotic effects on bacterial growth dynamics. The minimum inhibitory concentration (MIC) was defined as the lowest antibiotic concentration at which no measurable growth relative to the untreated control was observed under the experimental conditions used in this study. Because growth was monitored continuously in anaerobic WC medium rather than by the standardized CLSI broth microdilution endpoint procedure, these values should be interpreted as assay-specific MIC estimates. Values reported as “>” indicate that measurable growth was still observed at the highest concentration tested and that the true MIC exceeded the upper limit of the tested concentration range. Raw OD data were exported from the plate reader software for further analysis.

### Quantification of growth curve characteristics

We extracted the maximum and final OD, with associated error bars, directly from the experimental data. We estimated maximum and minimum slopes by computing the first derivative of interpolated OD curves using a Savitzky–Golay filter [55] with a linear model and a 10-hour sliding window. We quantified uncertainty in the slope estimates using the experimental replicate error estimates within the 10-hour window centered on the peak derivative.

We selected a 10-hour sliding window after evaluating window lengths between 5 and 20 h. Shorter windows produced slope estimates dominated by local fluctuations, whereas longer windows reduced sensitivity to biologically distinct growth and decline phases. A 10-hour window balanced noise suppression with preservation of the dominant dynamical features of the approximately 50-hour time series.

### Growth model fitting

We modelled bacterial growth dynamics using a Monod-type ordinary differential equation (ODE) system that includes substrate limitation, lag-phase adaptation, and a death term, with the equation of the lag phase inspired by [56]. The model describes the evolution of the substrate concentration *S * and bacterial density *X* according to

$$\frac{\mathrm{d}S\left(t\right)}{\mathrm{d}t}=-\frac{1}{Y}L\left(t\right)g\left(S\right)X\left(t\right)$$

$$\frac{\mathrm{d}X\left(t\right)}{\mathrm{d}t}=L\left(t\right)g\left(S\right)X\left(t\right)-d\:X\left(t\right),$$

where $g\left(S\right)=r\frac{S}{S+K}$ is the Monod growth function with maximum growth rate *r* and half-saturation constant *K*. We normalized substrate such that $S\left(0\right)=1$, rendering *S* and *K* dimensionless. Lag-phase dynamics are modeled through $L\left(t\right)=\frac{Q\left(t\right)}{1+Q\left(t\right)}$, with $Q\left(t\right)=0.1\:\mathrm{exp}\left(s\:t\right)$, where *s* is the lag adaptation rate. The parameter *d* represents the death rate and *Y* is the yield coefficient (substrate consumed per unit biomass produced). This model captures the characteristic phases of bacterial growth: an initial lag phase, exponential growth under substrate sufficiency, a stationary phase as substrates become limiting, and a subsequent decline. Although we did not measure the substrate in this work, we observed that glucose limits the growth for most of our strains in the same medium (for *B. thetaiotaomicron* and *R. intestinalis*, see [57] and unpublished data for *B. fragilis* and *E. coli* LF82), whereas *B. hydrogenotrophica*’s growth stops after depletion of trehalose and glucose [58]. We fixed *K=0.2* because parameter estimation was based on biomass data only. Sensitivity analysis for $K\in\:\left\{\mathrm{0.1,0.5}\right\}$ showed that the inferred parameter values remained consistent across the tested range.

Because substrates were not measured, the substrate variable represents a normalized latent resource rather than a specific nutrient. Hence, the model does not capture diauxic or biphasic growth, and the inferred growth rate $\:r\:$ and yield $\:Y\:$should be interpreted as effective parameters summarizing the overall growth characteristics rather than substrate- or phase-specific quantities.

We implemented the model using the Stan probabilistic programming language, which employs Hamiltonian Monte Carlo sampling with the No-U-Turn Sampler for efficient posterior exploration. Convergence of the Markov chains is assessed using the potential scale reduction factor $\hat{R}$, with values of $\hat{R}<1.01$ indicating convergence. We use data-informed lognormal priors for the kinetic parameters. That is,

$$\:r\sim\:\mathrm{Lognormal}\left(\mathrm{log}\left({r}_{\mathrm{est}}\right),1.0\right),\:$$

$$\:s\sim\:\mathrm{Lognormal}\left(\mathrm{log}\left({s}_{\mathrm{est}}\right),0.5\right),\:$$

$$d\sim\:\mathrm{Lognormal}\left(\mathrm{log}\left({d}_{\mathrm{est}}\right),0.5\right),$$

$$a\sim\:\mathrm{Lognormal}\left(\mathrm{log}\left({a}_{\mathrm{est}}\right),0.5\right),$$

where *a* denotes the inverse yield coefficient. The location parameters ${r}_{\mathrm{est}}$, ${s}_{\mathrm{est}}$, ${d}_{\mathrm{est}}$, and ${a}_{\mathrm{est}}$ are computed through a mechanistic analysis of growth-curve features, including the lag, growth, and decline phases. Lognormal priors were chosen to enforce positivity and to reflect multiplicative uncertainty in the rate parameters. We assign an exponential prior to the observation noise standard deviation, with a rate parameter based on the mean empirical standard deviation. We solve the ODE system numerically using the Dormand–Prince method, and the likelihood is modeled as a normal distribution.

We included parameter estimates in our results only for growth curves that reached a maximum OD of at least 0.1. Death rates were reported when the maximum OD occurred at least 10 h before the final measurement.

## Results

### Effects of low concentrations of antibiotics on the growth curves of gut bacterial species

**Fig. 1:**
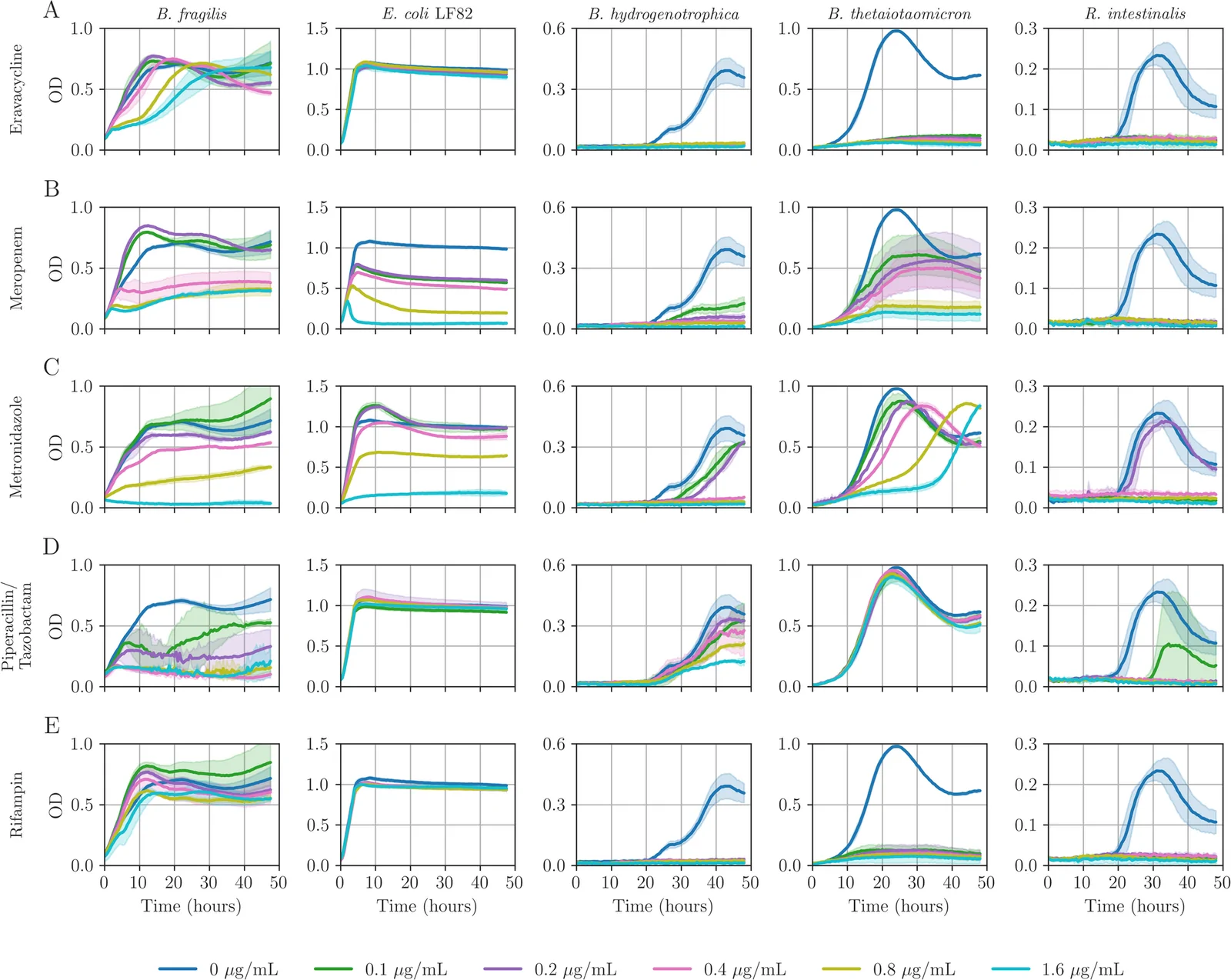
Low concentration antibiotic effects on gut bacterial growth dynamics. OD growth curves of five bacterial strains measured over time in the presence of five different concentrations of antibiotics. Rows correspond to antibiotics: eravacycline (**A**), meropenem (**B**), metronidazole (**C**), piperacillin/tazobactam (**D**) and rifampin (**E**). Columns correspond to bacterial members: *B. fragilis*, *E. coli* LF82, *B. thetaiotaomicron*, *B. hydrogenotrophica* and *R. intestinalis*. Solid lines represent mean ODs over time, and shaded regions indicate the corresponding standard deviations

**Fig. 2:**
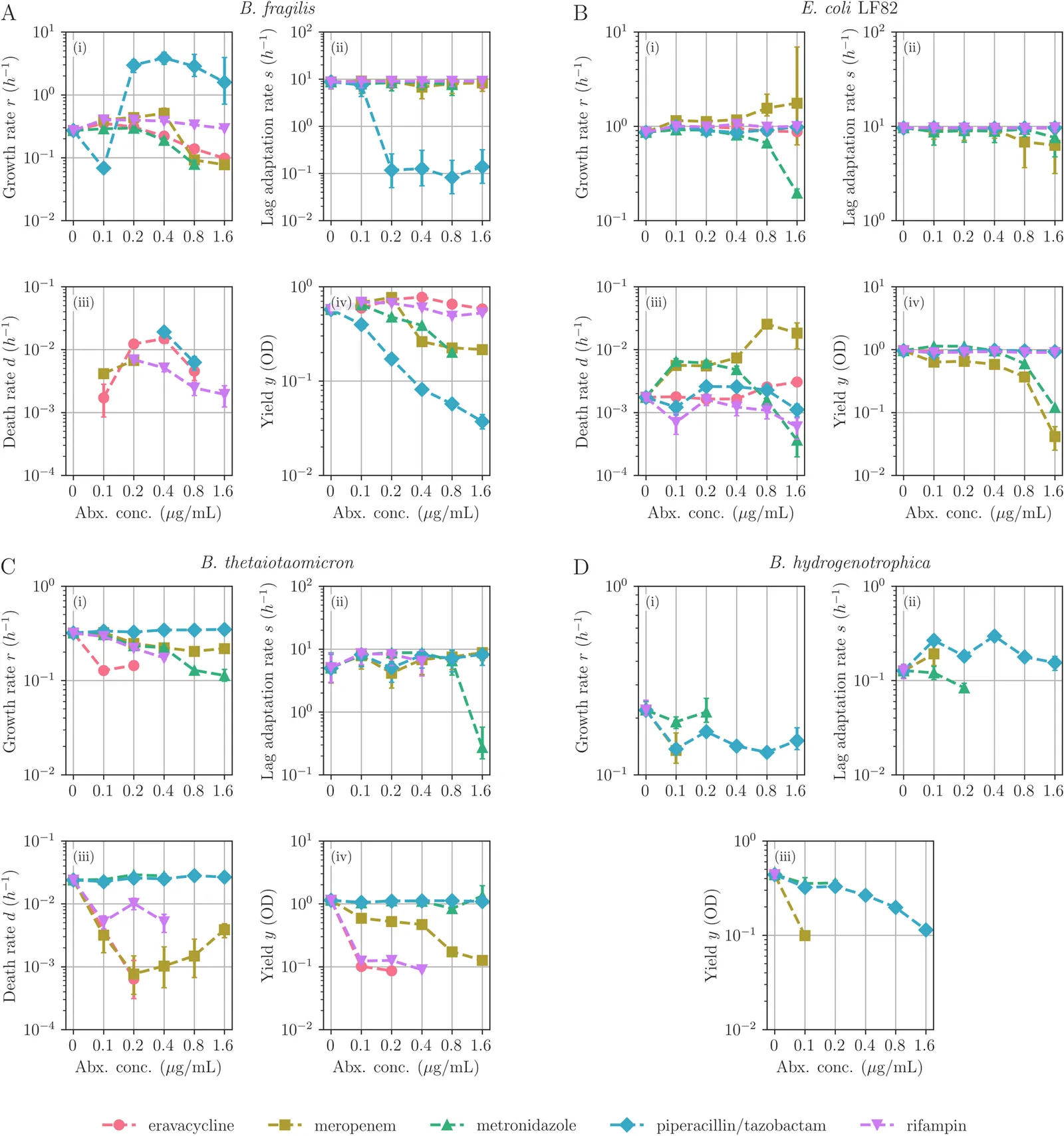
Alterations induced by antibiotics in bacterial growth curve parameters, which were obtained through model fitting. Parameters were quantified in the presence of increasing concentrations of five antibiotics for: *B. fragilis* (**A**), *E. coli* LF82 (**B**), *B. thetaiotaomicron* (**C**) and *B. hydrogenotrophica* (**D**). Panels show maximum growth rate (i), lag adaptation rate (ii), death rate (iii) and yield (iv), plotted as a function of the antibiotic concentration. Markers indicate median values, and error bars represent 90% credible intervals

We quantified the impact of the antibiotics on the growth of the selected gut bacterial strains for five concentrations in duplicate using the optimized plate reader set-up. The resulting growth curves are plotted in Fig. 1. We then fitted a Monod-type growth model to all growth curves with a corrected maximal OD equal to or above 0.1 to determine growth rates, death rates, yields and lags, summarized in Fig. 2. Since most *R. intestinalis* growth curves did not reach the minimum OD due to its high susceptibility to all tested antibiotics, the results presented below do not include *R. intestinalis*.

We found that metronidazole lowers the growth rate in a dose-dependent manner for all tested strains as well as their yields (except for *B. thetaiotaomicron*) but does not systematically increase the death rate (Fig. 2), which is in line with its mode of action, the inhibition of DNA synthesis (Table 1). Metronidazole resistance has been reported for both Bacteroides strains (Table 1), but their response to metronidazole does not differ much from that of the *E. coli* LF82 strain, which was not reported to harbor metronidazole resistance genes. Antibiotic resistance gene screening did not uncover any hits for the tested strains, and we thus assume that generic efflux mechanisms explain the observed resistances [59].

**Table 1:** Summary of antibiotic degradation and resistance genes identified by genome screening and reported in the literature for the studied gut bacterial strains. NCBI reference genome completeness assessed with CheckM [62] ranges from 99.61% for *Blautia hydrogenotrophica* DSM 10507 to 91.07% for *Roseburia intestinalis* L1-82

Antibiotic	Antibiotic class	Antibiotic mode of action	Strains	Resistance genes		Mechanism summary	References
				Detected through genome screening	Reported in literature		
Eravacycline	Tetracycline / fluorocycline	Binds 30S ribosomal subunit (specifically 16S rRNA) and blocks aminoacyl-tRNA binding, inhibits protein synthesis, mostly unaffected by common tetracycline resistance mechanisms [63]	*Escherichia coli* LF82	AcrAB-TolC (RND efflux pump); upregulated via AcrR mutation, MarR mutations, soxR/soxS regulons (tetracycline/glycylcycline/rifamycin class)	No tet-specific resistance genes reported	AcrAB-TolC upregulation via AcrR and MarR mutations, and via soxR/soxS regulons, reduces intracellular eravacycline concentrations by active efflux	[34, 64, 65]
			*Bacteroides fragilis* NCTC 9343	None detected	None known	—	—
			*Bacteroides thetaiotaomicron* VPI 5482	None detected	None known	—	—
			*Blautia hydrogenotrophica* DSM 10507	tet(32)	tet(32) in human gut Firmicutes	tet(32) encodes a GTP-dependent ribosomal protection protein that dislodges tetracycline from the 30S ribosomal unit, restoring protein synthesis without antibiotic degradation.	[66]
			*Roseburia intestinalis* L1-82	tet(O), tet(40)	tet(40) (MFS efflux pump)	tet(O) encodes a ribosomal protection protein; tet(40) encodes an MFS efflux pump; together they reduce tetracycline binding and intracellular antibiotic accumulation without drug degradation.	[14]
Meropenem	β-lactam (carbapenem)	Inhibits penicillin-binding proteins (PBPs), inhibits bacterial cell wall synthesis by blocking peptidoglycan cross-linking; stable towards most β-lactamases [67]	*Escherichia coli* LF82	No carbapenemase; AcrEF-TolC; EmrAB-TolC	Efflux systems reported in Enterobacterales	Multidrug efflux systems AcrEF–TolC and EmrAB–TolC reduce intracellular antibiotic concentrations by active export, contributing to decreased susceptibility to meropenem in the absence of carbapenemase-mediated degradation.	[31]
			*Bacteroides fragilis* NCTC 9343	None detected	None known	—	—
			*Bacteroides thetaiotaomicron* VPI 5482	None detected	None known	—	—
			*Blautia hydrogenotrophica* DSM 10507	None detected	None known	—	—
			*Roseburia intestinalis* L1-82	None detected	None known	—	—
Metronidazole	Nitroimidazole	Reduction of nitro group under anaerobic conditions activates the prodrug, nitro radicals interact with DNA and disrupt helical structure, leads to cell death in susceptible anaerobes [68]	*Escherichia coli* LF82	msbA (ABC efflux pump; nitroimidazole class)	None known (nim genes are rare in *E. coli*)	msbA encodes an ABC transporter that can export nitroimidazoles including metronidazole, reducing intracellular drug accumulation.	[21, 69]
			*Bacteroides fragilis* NCTC 9343	None detected	BmeRABC efflux system	BmeRABC is a multidrug efflux pump that actively exports metronidazole, reducing intracellular drug concentration. NCTC 9343 is confirmed nim-negative; observed resistance is likely efflux-mediated	[21,38, 59, 70]
			*Bacteroides thetaiotaomicron* VPI 5482	None detected	nim-like nitroimidazole reductase (5-nitroimidazole resistance protein)	nim-like nitroreductase can reduce the nitro group of metronidazole to inactive amine derivatives	[21, 43]
			*Blautia hydrogenotrophica* DSM 10507	None detected	None known	—	—
			*Roseburia intestinalis* L1-82	None detected	None known	—	—
Piperacillin/tazobactam	β-lactam (penicillin) + β-lactamase inhibitor	Piperacillin binds to penicillin-binding proteins (PBPs) and inhibits bacterial cell wall synthesis by blocking peptidoglycan cross-linking; Tazobactam inhibits several class A β-lactamases and protects piperacillin from hydrolysis when used in combination [71]	*Escherichia coli* LF82	EC-5 (EC β-lactamase), AcrEF-TolC, EmrAB-TolC	EC-5 (EC β-lactamase); efflux systems AcrEF-TolC, EmrAB-TolC	EC β-lactamase (EC-5) mediates hydrolysis of the β-lactam component, while multidrug efflux via AcrEF–TolC and EmrAB–TolC further reduces intracellular antibiotic levels, together contributing to resistance to piperacillin/tazobactam.	[33, 34]
			*Bacteroides fragilis* NCTC 9343	CepA-44 (serine β-lactamase)	CepA (class A β-lactamase)	CepA-44 hydrolyses the β-lactam ring of the penicillins and cephalosporins, reducing inhibition of penicillin-binding proteins.	[72]
			*Bacteroides thetaiotaomicron* VPI 5482	None detected	BT_4507, a chromosomal class A serine β-lactamase related to CepA	Chromosomal class A serine β-lactamase that hydrolyzes β-lactams; high expression can reduce the protection provided by tazobactam.	[61]
			*Blautia hydrogenotrophica* DSM 10507	None detected	None known	—	—
			*Roseburia intestinalis* L1-82	None detected	None known	—	—
Rifampin	Ansamycin	Binds to β-subunit of DNA-dependent RNA polymerase (rpoB) and inhibits DNA-dependent RNA synthesis [73]	*Escherichia coli* LF82	AcrAB-TolC; upregulated via AcrR mutation, soxR/soxS regulons	Not inherently present; might occur due to rpoB gene mutation	AcrR mutation and soxR/soxS regulon activation upregulate AcrAB-TolC expression, increasing efflux of rifampin; no inactivating enzyme detected; rpoB gene mutation causes decreased affinity to rifampin to its binding site.	[34, 74, 75]
			*Bacteroides fragilis* NCTC 9343	None detected	None known	—	—
			*Bacteroides thetaiotaomicron* VPI 5482	None detected	None known	—	—
			*Blautia hydrogenotrophica* DSM 10507	None detected	None known	—	—
			*Roseburia intestinalis* L1-82	None detected	None known	—	—

Meropenem increases the death rate in *E. coli* LF82 and *B. fragilis* and thus acts as a bactericide, in agreement with its mode of action (inhibition of cell wall synthesis). Meropenem did not consistently increase the death rate of *B. thetaiotaomicron* and its impact on the death rate of *B. hydrogenotrophica* could not be reliably determined, but in both type strains, the maximal OD and slope were lowered in a dose-dependent manner (Additional file 1: Supplementary Figure S3). Meropenem also decreases the yield in all strains and the growth rate in all strains except *E. coli* LF82. We did not find carbapenemases in the genome of *E. coli* LF82 (Table 1), but they were detected in other *E. coli* strains [60] and were reported to occur in clinical isolates of the Bacteroides fragilis group [41]. The observed partial meropenem resistance in *E. coli* LF82 can be explained by the presence of dedicated efflux pumps such as AcrEF-TolC and EmrAB-TolC (Table 1). Of note, we did not see an effect of meropenem on the growth rate or lag phase of *E. coli* LF82. Thus, the effect of resistance gene expression on its fitness is either below the detection limit, or *E. coli* expresses these genes constitutively.

*E. coli* LF82 and the type strain of *B. thetaiotaomicron* are resistant to the combination of piperacillin and tazobactam, which does not significantly affect their growth curve parameters compared to the control. They were also both reported to encode β-lactamases that confer resistance to piperacillin/tazobactam [33, 42, 61], although we did not detect a β-lactamase in *B. thetaiotaomicron*’s genome (Table 1). Of note, the growth curves of *B. hydrogenotrophica* are not as strongly affected by this combination than those of *B. fragilis*, though no resistance genes to piperacillin/tazobactam were reported or found in the *B. hydrogenotrophica* type strain.

Rifampin does not affect the growth curve parameters of *E. coli* LF82 and has variable effects on the growth and death rates of *B. fragilis* and *B. thetaiotaomicron*. In contrast to *B. fragilis*, the growth curve parameters of the type strain of *B. thetaiotaomicron* (in particular yield) were affected by rifampin. This difference in the response of *B. fragilis* and *B. thetaiotaomicron* to rifampin could not be explained based on the antibiotic resistance genes detected in their genomes.

Eravacycline reduces the growth rate of *B. thetaiotaomicron* and *B. fragilis* type strains in agreement with its mode of action, i.e. blocking protein synthesis. Although no enzyme-encoding resistance gene was reported or detected for *E. coli* LF82, the strain displays eravacycline resistance, which increases its death rate only at higher concentrations but does not affect its growth rate. This resistance is likely conferred by efflux pumps. Eravacycline resistance is also increasingly reported in clinical isolates of *E. coli* [64, 65].

To complement our characterization of growth dynamics, we also estimated the effective growth rate as the maximum slope of the measured growth curves (after smoothing) and checked if these rates show the same tendency as the growth rates *r* estimated from model fitting. Note that this effective rate is not expected to quantitatively match *r*. Growth rate and maximal slope were in most cases well correlated, with the exception of *E. coli* LF82, which has the highest growth rate among all tested strains and thus the fewest measurement points available to estimate the positive slope. When it was possible to compare death rate and negative slope across a range of concentrations (for *B. thetaiotaomicron* and *E. coli* LF82), they were also well correlated.

Table 2 summarizes the assay-specific MIC estimates obtained from the growth-curve experiments and compares them with previously reported strain-specific MIC values or CLSI quality-control ranges where available. Only strain-specific literature MIC values or strain-specific quality control (QC) ranges were included for comparison with MIC values obtained from this study. Overall, meropenem is the most effective antibiotic, increasing the death rate of the pathogenic *E. coli* LF82 and lowering the growth rate of both Bacteroides type strains but it also affects the commensal strains.

**Table 2:** Comparison of MICs determined in this study with previously reported strain-specific values or reference quality-control (QC) ranges. A graphical summary is provided in Additional file 1: Supplementary Figure S9. Differences should be interpreted with caution because cultivation conditions, media composition, incubation procedures and endpoint determination differ from standardized CLSI susceptibility testing methods. Values reported as “>” indicate that no inhibitory endpoint was reached within the tested concentration range and that the true MIC exceeded the highest concentration tested

Antibiotic	Strains	Highest concentration tested (µg/mL)	MIC in this study (µg/mL)	Previously reported MIC or QC range (µg/mL)	References
Eravacycline	*Escherichia coli* LF82	16	1.6	No strain-specific MIC reported	—
	*Bacteroides fragilis* NCTC 9343	16	1.6	CLSI QC MIC range: 0.06–0.25 (agar dilution) or 0.016–0.12 (broth microdilution)	[48]
	*Bacteroides thetaiotaomicron* VPI 5482	16	0.4	No strain-specific MIC reported	—
	*Roseburia intestinalis* L1-82	1.6	0.1	No strain-specific MIC reported	—
	*Blautia hydrogenotrophica* DSM 10507	1.6	0.1	No strain-specific MIC reported	—
Meropenem	*Escherichia coli* LF82	2	2^b^	No strain-specific MIC reported	—
	*Bacteroides fragilis* NCTC 9343	16	2	CLSI QC MIC range: 0.03–0.25 µg/mL (for both agar and broth dilution method)	[48]
	*Bacteroides thetaiotaomicron* VPI 5482	16	1	MIC: 0.125-0.5 (agar dilution)	[30]
	*Roseburia intestinalis* L1-82	1.6	0.1	MIC: 0.023–0.064 (agar dilution)	[30]
	*Blautia hydrogenotrophica* DSM 10507	1.6	0.4	No strain-specific MIC reported	—
Metronidazole	*Escherichia coli* LF82	1.6	> 1.6^a^	No strain-specific MIC reported	—
	*Bacteroides fragilis* NCTC 9343	16	16	CLSI QC MIC range: 0.25 − 1 (agar dilution) or 0.25 − 2(broth microdilution)	[48]
	*Bacteroides thetaiotaomicron* VPI 5482	16	16	MIC 16 (agar dilution)	[30]
	*Roseburia intestinalis* L1-82	1.6	0.1	0.5–0.75 (agar dilution)	[30]
	*Blautia hydrogenotrophica* DSM 10507	1.6	0.4	No strain-specific MIC reported	—
Piperacillin/tazobactam	*Escherichia coli* LF82	16	> 16/4^a^	No strain-specific MIC reported	—
	*Bacteroides fragilis* NCTC 9343	16	16/4	CLSI QC MIC range: 0.125/4 − 0.5/4 (agar dilution) or 0.03/4 − 0.25/4 (broth microdilution)	[48]
	*Bacteroides thetaiotaomicron* VPI 5482	16	> 16/4^a^	No strain-specific MIC reported	—
	*Roseburia intestinalis* L1-82	1.6	0.2/0.4	No strain-specific MIC reported	—
	*Blautia hydrogenotrophica* DSM 10507	1.6	> 1.6/0.4^a^	No strain-specific MIC reported	—
Rifampin	*Escherichia coli* LF82	16	> 16^a^	No strain-specific MIC reported	—
	*Bacteroides fragilis* NCTC 9343	16	2	No strain-specific MIC reported	—
	*Bacteroides thetaiotaomicron* VPI 5482	16	1.6	No strain-specific MIC reported	—
	*Roseburia intestinalis* L1-82	1.6	0.1	No strain-specific MIC reported	—
	*Blautia hydrogenotrophica* DSM 10507	1.6	0.1	No strain-specific MIC reported	—

^a^Endpoint not reached at highest tested concentration; a dose-response plateau was observed, suggesting the strain is resistant or tolerant at the tested concentrations

^b^Meropenem MIC for *E. coli* LF82 determined from a dedicated run (See additional file 1: Supplementary Figure S8)

### Effects of high concentrations of antibiotics on the growth curves of selected gut bacterial species

**Fig. 3:**
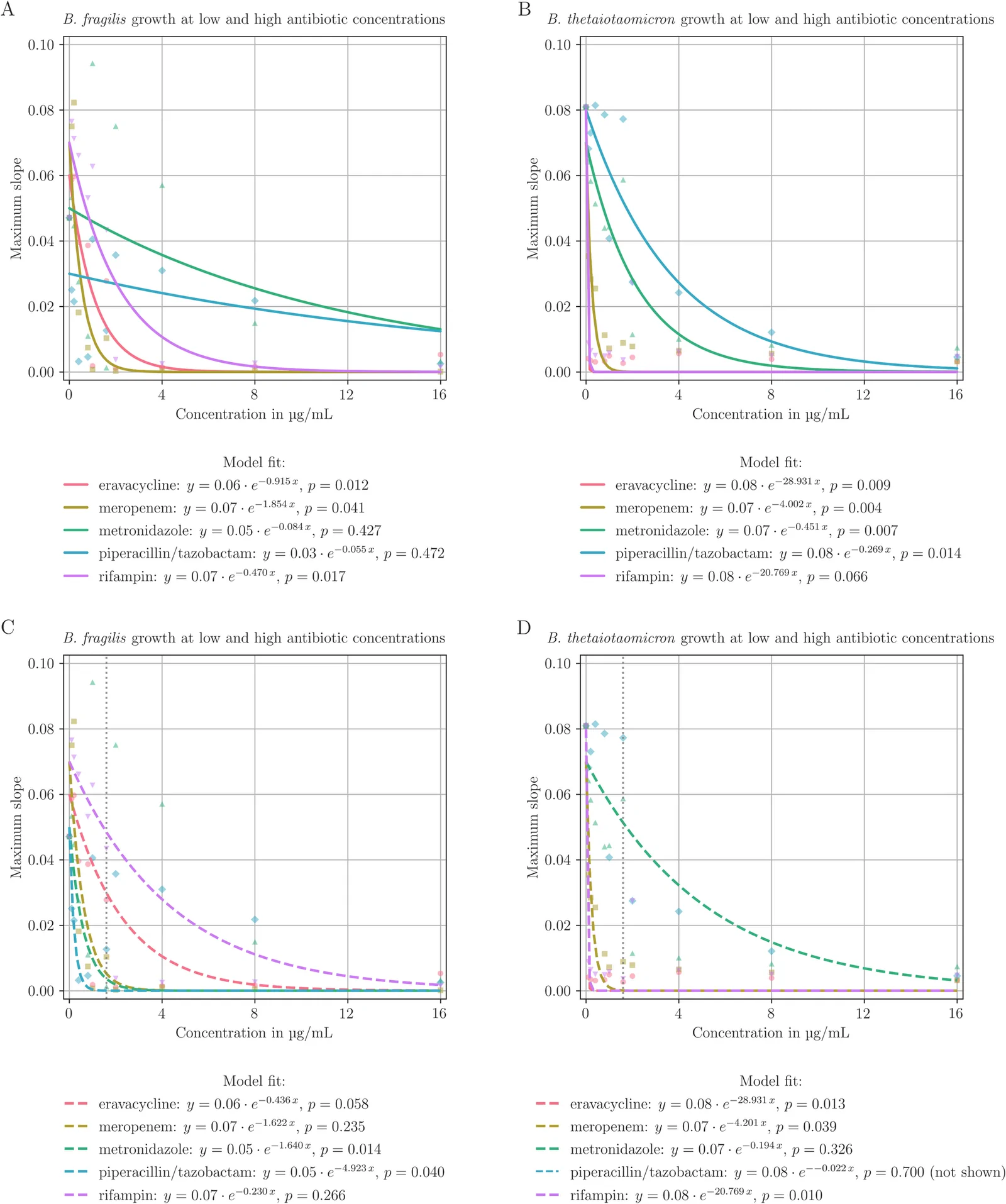
Maximum slopes decay exponentially with antibiotic concentration. **A**, **B**: Exponential decay fitted to maximum slopes of growth curves in low and high antibiotic concentration ranges for the type strains of *B. fragilis* (**A**) and *B. thetaiotaomicron* (**B**). **C**, **D**: Exponential decay curves were derived only based on maximum slopes at low concentrations and extrapolated to high concentrations for *B. fragilis* (**C**) and *B. thetaiotaomicron* (**D**). Fitting was carried out with non-linear least squares implemented using the R function *nls*

*E. coli* LF82 and the type strains of *B. thetaiotaomicron* and *B. fragilis* displayed resistance to several antibiotics in the low concentration range. We therefore tested the response to higher concentrations of all five antibiotics for *B. thetaiotaomicron* and *B. fragilis* (Fig. 3 and Additional file 1: Supplementary Figure S4) and to higher concentrations of eravacycline, piperacillin/tazobactam and rifampin for *E. coli* LF82 (Additional file 1: Supplementary Figure S4). We then selected the maximum slope as a characteristic that is easily derived from the growth curves and well correlated to the growth rate obtained through model fitting (Additional file 1: Supplementary Figure S6). We found that exponential decay well describes the observed change in maximum slopes for the two Bacteroides species across both low and high concentrations for all antibiotics, except for metronidazole and piperacillin/tazobactam in *B. fragilis* (Fig. 3A, B). In some cases, the non-linear relationship derived for maximum slopes at low concentrations also predicts well the maximum slopes detected at high concentrations (Fig. 3C, D).

### CFU counts confirm dose-dependent bactericidal activity of meropenem

To directly validate that the meropenem-associated death rate parameter inferred from OD-based model fitting reflects true loss of cell viability, we counted colony-forming units (CFUs) for *E. coli* LF82 and *B. fragilis* NCTC 9343 for two time points (8 h and 24 h) and three conditions (control, 0.2 µg/mL and 2.0 µg/mL meropenem), with six biological replicates per condition (Additional file 1: Supplementary Figure S7).

In addition to the CFU validation, a validation experiment using six biological replicates per strain qualitatively reproduced the concentration-dependent growth responses observed in the original dataset despite batch effects (Additional file 1: Supplementary Figure S8).

Linear mixed-effects model analysis confirmed that meropenem concentration had a highly significant effect on log₁₀ CFU/mL in both *B. fragilis* (F = 142.4, df = 2/169, *p* < 2.2 × 10⁻¹⁶) and *E. coli* LF82 (F = 256.1, df = 2/174, *p* < 2.2 × 10⁻¹⁶). Timepoint alone was not significant as a main effect for either species (*B. fragilis*: *p* = 0.534; *E. coli* LF82: *p* = 0.717), reflecting the dominant contribution of concentration to the observed variation in viable counts. The condition-by-timepoint interaction was significant for both species (*B. fragilis*: F = 10.09, *p* = 7.28 × 10⁻⁵; *E. coli* LF82: F = 16.96, *p* = 1.86 × 10⁻⁷), indicating that the magnitude of meropenem-induced reduction in viable cell counts increases with time, consistent with the death rate estimates.

Polynomial contrast analysis of estimated marginal means confirmed a significant negative linear trend across ordered concentration levels (control → low → high) at both timepoints and in both species (all *p* ≤ 0.003), consistent with dose-dependent reduction in viable cell counts. For *B. fragilis*, the linear contrast estimate was − 8.57 (SE = 1.04, *p* < 0.0001) at T = 8 h and − 14.55 (SE = 1.04, *p* < 0.0001) at T = 24 h, indicating a 1.7-fold greater concentration-dependent reduction in viable cell counts between the two timepoints. For *E. coli* LF82, the linear contrast estimate was − 10.41 (SE = 0.888, *p* < 0.0001) at T = 8 h and − 16.02 (SE = 0.888, *p* < 0.0001) at T = 24 h. Significant quadratic contrasts at both timepoints and in both species (all *p* ≤ 0.003) indicate that the dose-response is non-linear on the log scale, with a disproportionately large decrease in viable counts between the control and low concentration condition relative to the additional reduction from low to high, consistent with strong bactericidal action near the MIC. Together, these results confirm concentration- and time-dependent bactericidal activity by meropenem in both species.

## Discussion

Here, we investigated the response of five gut bacterial strains to five commonly used antibiotics by quantifying their effects on different growth characteristics. To this end, we employed a generic growth model that assumes the consumption of a single carbon source, which is justified based on data we collected previously for these species in WC medium (for *B. thetaiotaomicron*, *B. hydrogenotrophica* and *R. intestinalis*, see [57, 58]; unpublished data for *B. fragilis* and *E. coli* LF82). This simple model fitted most growth curves with high goodness of fit (Additional file 1: Supplementary Figure S2). However, there were a few notable exceptions. For instance, the growth model assumes a constant death rate. This assumption may be violated in the case of β-lactam antibiotics, which interfere with cell wall synthesis. β-lactam antibiotics are known to trigger autolysis in *E. coli* [76, 77] through an imbalance of peptidoglycan-degrading autolysins and their regulators. The resulting accelerated death rate is not accounted for in the model and may explain the dose-dependent deterioration of the goodness of fit for meropenem in *E. coli* LF82. In addition, in *B. fragilis* and *B. thetaiotaomicron*, there are several instances where a rapid initial increase of OD is followed by a decline and a second, slower, increase. This biphasic growth is observed in the control as well as for low concentrations of all five antibiotics and may indicate a switch to another nutrient in a diauxic shift. In the case of *B. thetaiotaomicron*, the new nutrient is likely mannose, which is present in low concentrations in the medium [57]. The model only accounts for a single nutrient, and thus diauxic shifts are not correctly described. In a few cases in *B. fragilis*, the switch to the second growth phase occurs much earlier than in the control. This was observed for the lowest concentration of piperacillin/tazobactam and for intermediate concentrations of metronidazole and meropenem. In these cases, biphasic growth is likely not triggered by nutrient depletion but could be due to a change in gene expression triggered by accumulating physiological effects of antibiotic exposure.

We also note that antibiotic resistances expected based on genome screening were not always in agreement with observations. For example, *B. fragilis*’ partial resistance to rifampin was not expected based on its antibiotic resistance gene (ARG) profile and *B. thetaiotaomicron* with a similar ARG profile was sensitive to it. Likewise, piperacillin/tazobactam resistance, in particular for *B. hydrogenotrophica*, was hard to explain based on ARG profiles. Furthermore, there were also contradictions between strain-specific antibiotic resistances reported in the literature and genome screening results. These deviations may be explained by the presence of unknown or strongly modified resistance genes or by recently acquired resistance genes (e.g. on plasmids) that are not present in the screened genome or by short-term evolution of resistance during the experiment [78]. Thus, ARG screening of genomes is not sufficient to predict antibiotic resistances.

Overall, we found that broad-spectrum antibiotics such as meropenem can successfully suppress a pathogenic strain involved in IBD but at the cost of affecting commensal strains as well. This finding agrees with the ambiguous outcomes reported for broad-spectrum antibiotic treatment of IBD [79, 80]. Our results also illustrate that commensal strains from culture collections can harbor resistances against popular antibiotics, such as *B. hydrogenotrophica* against piperacillin/tazobactam. Thus, commensal strains that are otherwise beneficial to the host can serve as antibiotic resistance reservoirs, which is also evidenced by the wide distribution of ARGs found in human gut microbiota [81, 82]. In addition, *E. coli* LF82 is resistant to two of the tested antibiotics even at high concentrations (rifampin and piperacillin/tazobactam), which means that these antibiotics not only harm commensals such as *R. intestinalis* but are also not effective against their target organisms.

We also saw that growth and death rates obtained by fitting a growth model are well correlated to the minimum and maximum slopes. This means that MIC50 values can be defined in terms of the maximum slope as a proxy for growth rate rather than the area under the growth curve, which is dependent on experiment duration. In addition, we observed that the exponential decay of maximum slopes with increasing antibiotic concentrations was in some cases predictive for maximum slopes recorded for higher concentrations, which means that MIC50 values defined based on growth rates can be extrapolated in these cases.

We only tested a single strain per species in an artificial environment in monoculture. Given that antibiotic resistances can vary widely across strains of the same species (e.g. [83]). and that in vivo conditions differ from those in vitro, these findings cannot be generalized to species level or to the gut environment. Nevertheless, our observations are in line with a meta-study [84] that reported a higher level of ARGs in Bacteroidia (including *B. fragilis* and *B. thetaiotaomicron*) and Gammaproteobacteria (including *E. coli*) than other gut bacterial classes in fecal samples. Our findings also agree with the consensus that broad-spectrum antibiotics negatively affect critical functions in the gut microbiome, such as butyrate production [85], and that thus more targeted approaches are required to eradicate pathogens while sparing commensals [86]. Narrow-spectrum antimicrobials such as ridinilazole and thuricine CD have been successfully employed against pathogenic *Clostridioides difficile* strains [87, 88]. In addition, a new bacteriocin, enterololin, was developed against adherent-invasive *E. coli* strains [89]. Another strategy is to provide antidotes that mitigate the effect of antibiotics on commensals, as recently shown in the case of erythromycin combined with the antidote dicumarol, which killed the pathogen *E. faecalis* but spared commensal Bacteroides species [30].

CFU counts analyzed using a linear mixed-effects model confirmed that meropenem induces significant, concentration-dependent reduction in viable cell counts in both *E. coli* LF82 and *B. fragilis* NCTC 9343. The significant condition-by-timepoint interaction demonstrates that the meropenem-induced bactericidal effect increases with time, consistent with the OD-derived death rate estimates. In *B. thetaiotaomicron*, we previously observed a constant total cell count for several days after nutrient depletion, accompanied by a drastic drop in the number of live cells [57]. Thus, we interpreted death rates derived from OD measurements with caution. In addition, since low- and high antibiotic concentrations were tested separately, batch effects affect the comparison of these two concentration ranges. Furthermore, we did not test all antibiotics relevant for IBD treatment, such as ciprofloxacin or rifaximin [80].

We did not find substantial differences between growth curve characteristics of species exposed to antibiotics that are inactivated (degraded, e.g. piperacillin by β-lactamase) and exposed to those that are not altered (e.g. rifampin, for which there is no known resistance in our strains). Previous work shows that the susceptibility of *E. coli* MG1655 to antibiotics depends on its growth rate, which was varied by supplying nutrients of different quality to the medium [90]. The susceptibility to irreversibly binding ribosome-targeting antibiotics was then seen to decrease with increasing nutrient quality, which was not the case for reversibly binding ribosome-targeting antibiotics, since their concentration is not reduced by increasing cell numbers. This relationship may hold in general for antibiotics that affect growth rates and are altered by a resistance mechanism, which would be interesting to investigate in the future.

Finally, we did not test the impact of other gut microbial strains on the response of the tested strains to the antibiotics. Previous work showed that species interactions can enhance or lower the sensitivity of gut bacterial species to antibiotics [91, 92]. Thus, investigating antibiotic responses in a community context is the next step.

## Conclusions

Our observation that the butyrate producer *R. intestinalis* L1-82 and the acetogen *B. hydrogenotrophica* DSM 10507 were more sensitive to the tested antibiotics than the *B. fragilis* type strain and the pathogen *E. coli* LF82 supports the hypothesis that broad-spectrum antibiotics deplete commensals while not necessarily being effective against opportunistic and pathogenic strains found in dysbiotic gut microbiota in IBD.

## Supplementary Information

Supplementary Material 1: Supplementary figures supporting the main manuscript, including additional growth curves, model diagnostics and comparisons between growth-curve characteristics, model-derived parameters, CFU counts and MIC data. Supplementary tables providing supporting information for 16S rRNA gene sequencing for quality control and whole genome sequencing analysis for *E. coli* LF82.

Supplementary Material 2: Raw growth curve dataset. Raw optical density measurements and associated data used for the growth curve analyses in this study.

Supplementary Material 3: Raw CFU counts for death rate validation for meropenem: Raw colony count data and associated data from the meropenem CFU validation experiment with *B. fragilis* NCTC 9343 and *E. coli* LF82.

Supplementary Material 4: Raw growth curve dataset for replicate validation under meropenem treatment. Raw optical density measurements and associated data from the additional six-replicate meropenem validation experiment with *B. fragilis* and *E. coli* LF82.

## Data Availability

All data generated or analyzed during this study are included in the manuscript and its supplementary information. Supplementary figures and tables are provided in Additional file 1. Raw growth curve data are available in Additional files 2 and 4. Raw CFU count data for meropenem death rate validation experiment are available in Additional file 3.

## References

[1] The Human Microbiome Project Consortium. Structure, function and diversity of the healthy human microbiome. Nature. 2012;486:207–14. 10.1038/nature11234. PMC3564958 22699609

[2] Sonnenburg JL, Bäckhed F. Diet–microbiota interactions as moderators of human metabolism. Nature. 2016;535:56–64. 10.1038/nature18846. PMC5991619 27383980

[3] Arumugam M, Raes J, Pelletier E, Le Paslier D, Yamada T, Mende DR, et al. Enterotypes of the human gut microbiome. Nature. 2011;473:174–80. 10.1038/nature09944. PMC3728647 21508958

[4] Costea PI, Hildebrand F, Arumugam M, Bäckhed F, Blaser MJ, Bushman FD, et al. Enterotypes in the landscape of gut microbial community composition. Nat Microbiol. 2017;3:8–16. 10.1038/s41564-017-0072-8. PMC5832044 29255284

[5] Lloyd-Price J, Arze C, Ananthakrishnan AN, Schirmer M, Avila-Pacheco J, Poon TW, et al. Multi-omics of the gut microbial ecosystem in inflammatory bowel diseases. Nature. 2019;569:655–62. 10.1038/s41586-019-1237-9. PMC6650278 31142855

[6] Franzosa EA, Sirota-Madi A, Avila-Pacheco J, Fornelos N, Haiser HJ, Reinker S, et al. Gut microbiome structure and metabolic activity in inflammatory bowel disease. Nat Microbiol. 2018;4:293–305. 10.1038/s41564-018-0306-4. PMC6342642 30531976

[7] Becker HEF, Jamin C, Bervoets L, Boleij A, Xu P, Pierik MJ, et al. Higher Prevalence of Bacteroides fragilis in Crohn’s Disease Exacerbations and Strain-Dependent Increase of Epithelial Resistance. Front Microbiol. 2021;12:598232. 10.3389/fmicb.2021.598232. PMC8219053 34168621

[8] Pascal V, Pozuelo M, Borruel N, Casellas F, Campos D, Santiago A, et al. A microbial signature for Crohn’s disease. Gut. 2017;66:813–22. 10.1136/gutjnl-2016-313235. PMC5531220 28179361

[9] Metwaly A, Dunkel A, Waldschmitt N, Raj ACD, Lagkouvardos I, Corraliza AM, et al. Integrated microbiota and metabolite profiles link Crohn’s disease to sulfur metabolism. Nat Commun. 2020;11:4322. 10.1038/s41467-020-17956-1. PMC7456324 32859898

[10] Núñez-Sánchez MA, Melgar S, O’Donoghue K, Martínez-Sánchez MA, Fernández-Ruiz VE, Ferrer-Gómez M, et al. Crohn’s Disease, Host–Microbiota Interactions, and Immunonutrition: Dietary Strategies Targeting Gut Microbiome as Novel Therapeutic Approaches. IJMS. 2022;23:8361. 10.3390/ijms23158361. PMC9369148 35955491

[11] Rashed R, Valcheva R, Dieleman LA. Manipulation of Gut Microbiota as a Key Target for Crohn’s Disease. Front Med. 2022;9:887044. 10.3389/fmed.2022.887044. PMC9244564 35783604

[12] Hodgkinson K, El Abbar F, Dobranowski P, Manoogian J, Butcher J, Figeys D, et al. Butyrate’s role in human health and the current progress towards its clinical application to treat gastrointestinal disease. Clin Nutr. 2023;42:61–75. 10.1016/j.clnu.2022.10.024. 36502573

[13] Sun X, Zhang Z, Hu J. Isolation, probiotic characterization and whole-genome sequencing of gut Faecalibacterium prausnitzii. Hum Nutr Metabolism. 2025;40:200315. 10.1016/j.hnm.2025.200315.

[14] Zhang C, Ma K, Nie K, Deng M, Luo W, Wu X, et al. Assessment of the safety and probiotic properties of Roseburia intestinalis: A potential Next Generation Probiotic. Front Microbiol. 2022;13:973046. 10.3389/fmicb.2022.973046. PMC9493362 36160246

[15] Kostic AD, Xavier RJ, Gevers D. The Microbiome in Inflammatory Bowel Disease: Current Status and the Future Ahead. Gastroenterology. 2014;146:1489–99. 10.1053/j.gastro.2014.02.009. PMC4034132 24560869

[16] Britton GJ, Contijoch EJ, Mogno I, Vennaro OH, Llewellyn SR, Ng R, et al. Microbiotas from Humans with Inflammatory Bowel Disease Alter the Balance of Gut Th17 and RORγt+ Regulatory T Cells and Exacerbate Colitis in Mice. Immunity. 2019;50:212–e2244. 10.1016/j.immuni.2018.12.015. PMC6512335 30650377

[17] D’Haens GR, Geboes K, Peeters M, Baert F, Penninckx F, Rutgeerts P. Early lesions of recurrent Crohn’s disease caused by infusion of intestinal contents in excluded ileum. Gastroenterology. 1998;114:262–7. 10.1016/S0016-5085(98)70476-7. 9453485

[18] Saint-Antoine IBD, Network, Sokol H, Landman C, Seksik P, Berard L, Montil M, et al. Fecal microbiota transplantation to maintain remission in Crohn’s disease: a pilot randomized controlled study. Microbiome. 2020;8:12. 10.1186/s40168-020-0792-5. PMC6998149 32014035

[19] Barnich N, Darfeuille-Michaud A. Adherent-invasive Escherichia coli and Crohnʼs disease: Current Opinion in Gastroenterology. 2007;23:16–20. 10.1097/MOG.0b013e3280105a38. 17133079

[20] Sprockett D, Fischer N, Boneh RS, Turner D, Kierkus J, Sladek M, et al. Treatment-Specific Composition of the Gut Microbiota Is Associated With Disease Remission in a Pediatric Crohn’s Disease Cohort. Inflamm Bowel Dis. 2019;25:1927–38. 10.1093/ibd/izz130. PMC7185687 31276165

[21] Löfmark S, Edlund C, Nord CE. Metronidazole Is Still the Drug of Choice for Treatment of Anaerobic Infections. CLIN INFECT DIS. 2010;50:S16–23. 10.1086/647939. 20067388

[22] Ianiro G, Tilg H, Gasbarrini A. Antibiotics as deep modulators of gut microbiota: between good and evil. Gut. 2016;65:1906–15. 10.1136/gutjnl-2016-312297. 27531828

[23] Patton PH, Parker CE, MacDonald JK, Chande N. Anti-tuberculous therapy for maintenance of remission in Crohn’s disease. Cochrane Database Syst Reviews. 2016;2016. 10.1002/14651858.CD000299.pub3. PMC6457855 27444319

[24] Mazuski JE, Tessier JM, May AK, Sawyer RG, Nadler EP, Rosengart MR, et al. The Surgical Infection Society Revised Guidelines on the Management of Intra-Abdominal Infection. Surg Infect. 2017;18:1–76. 10.1089/sur.2016.261. 28085573

[25] Solomkin JS, Yellin AE, Rotstein OD, Christou NV, Dellinger EP, Tellado JM, et al. Ertapenem Versus Piperacillin/Tazobactam in the Treatment of Complicated Intraabdominal Infections: Results of a Double-Blind, Randomized Comparative Phase III Trial. Ann Surg. 2003;237:235–45. 10.1097/01.SLA.0000048551.32606.73. PMC1522141 12560782

[26] Lowe MN, Lamb HM. Meropenem: An Updated Review of its Use in the Management of Intra-Abdominal Infections. Drugs. 2000;60:619–46. 10.2165/00003495-200060030-00010. 11030471

[27] Solomkin J, Evans D, Slepavicius A, Lee P, Marsh A, Tsai L, et al. Assessing the Efficacy and Safety of Eravacycline vs Ertapenem in Complicated Intra-abdominal Infections in the Investigating Gram-Negative Infections Treated With Eravacycline (IGNITE 1) Trial: A Randomized Clinical Trial. JAMA Surg. 2017;152:224. 10.1001/jamasurg.2016.4237. 27851857

[28] Solomkin JS, Gardovskis J, Lawrence K, Montravers P, Sway A, Evans D, et al. IGNITE4: Results of a Phase 3, Randomized, Multicenter, Prospective Trial of Eravacycline vs Meropenem in the Treatment of Complicated Intraabdominal Infections. Clin Infect Dis. 2019;69:921–9. 10.1093/cid/ciy1029. PMC6735687 30561562

[29] Ursing B, Kamme C, METRONIDAZOLE FOR CROHN’S DISEASE. Lancet. 1975;305:775–7. 10.1016/S0140-6736(75)92438-1. 48003

[30] Maier L, Goemans CV, Wirbel J, Kuhn M, Eberl C, Pruteanu M, et al. Unravelling the collateral damage of antibiotics on gut bacteria. Nature. 2021;599:120–4. 10.1038/s41586-021-03986-2. PMC7612847 34646011

[31] Demarre G, Prudent V, Schenk H, Rousseau E, Bringer M-A, Barnich N, et al. The Crohn’s disease-associated Escherichia coli strain LF82 relies on SOS and stringent responses to survive, multiply and tolerate antibiotics within macrophages. PLoS Pathog. 2019;15:e1008123. 10.1371/journal.ppat.1008123. PMC6855411 31725806

[32] Prudent V, Demarre G, Vazeille E, Wery M, Quenech’Du N, Ravet A, et al. The Crohn’s disease-related bacterial strain LF82 assembles biofilm-like communities to protect itself from phagolysosomal attack. Commun Biol. 2021;4:627. 10.1038/s42003-021-02161-7. PMC8149705 34035436

[33] Martinez-Medina M, Strozzi F, Del Ruiz B, Serrano-Morillas N, Ferrer Bustins N, Martínez-Martínez L. Antimicrobial Resistance Profiles of Adherent Invasive Escherichia coli Show Increased Resistance to β-Lactams. Antibiotics. 2020;9:251. 10.3390/antibiotics9050251. PMC7277491 32414140

[34] Fanelli G, Pasqua M, Prosseda G, Grossi M, Colonna B. AcrAB efflux pump impacts on the survival of adherent-invasive Escherichia coli strain LF82 inside macrophages. Sci Rep. 2023;13:2692. 10.1038/s41598-023-29817-0. PMC9931695 36792672

[35] Subramanian S, Roberts CL, Hart CA, Martin HM, Edwards SW, Rhodes JM, et al. Replication of Colonic Crohn’s Disease Mucosal *Escherichia coli* Isolates within Macrophages and Their Susceptibility to Antibiotics. Antimicrob Agents Chemother. 2008;52:427–34. 10.1128/AAC.00375-07. PMC2224732 18070962

[36] Wexler HM. *Bacteroides*: the Good, the Bad, and the Nitty-Gritty. Clin Microbiol Rev. 2007;20:593–621. 10.1128/CMR.00008-07. PMC2176045 17934076

[37] Snydman DR, Jacobus NV, McDermott LA, Ruthazer R, Golan Y, Goldstein EJC, et al. National Survey on the Susceptibility of *Bacteroides fragilis* Group: Report and Analysis of Trends in the United States from 1997 to 2004. Antimicrob Agents Chemother. 2007;51:1649–55. 10.1128/AAC.01435-06. PMC1855532 17283189

[38] Pumbwe L, Wareham DW, Aduse-Opoku J, Brazier JS, Wexler HM. Genetic analysis of mechanisms of multidrug resistance in a clinical isolate of Bacteroides fragilis. Clin Microbiol Infect. 2007;13:183–9. 10.1111/j.1469-0691.2006.01620.x. 17328731

[39] Rong SMM, Rodloff AC, Stingu C-S. Diversity of antimicrobial resistance genes in *Bacteroides* and *Parabacteroides* strains isolated in Germany. J Global Antimicrob Resist. 2021;24:328–34. 10.1016/j.jgar.2021.01.007. 33508481

[40] Jasemi S, Emaneini M, Ahmadinejad Z, Fazeli MS, Sechi LA, Sadeghpour Heravi F, et al. Antibiotic resistance pattern of Bacteroides fragilis isolated from clinical and colorectal specimens. Ann Clin Microbiol Antimicrob. 2021;20:27. 10.1186/s12941-021-00435-w. PMC8066845 33892721

[41] Wallace MJ, Jean S, Wallace MA, Burnham C-AD, Dantas G. Comparative Genomics of Bacteroides fragilis Group Isolates Reveals Species-Dependent Resistance Mechanisms and Validates Clinical Tools for Resistance Prediction. mBio. 2022;13:e03603–21. 10.1128/mbio.03603-21. PMC8764542 35038926

[42] Nagy E, Urbán E, Nord CE. Antimicrobial susceptibility of Bacteroides fragilis group isolates in Europe: 20 years of experience. Clin Microbiol Infect. 2011;17:371–9. 10.1111/j.1469-0691.2010.03256.x. 20456453

[43] Sadarangani SP, Cunningham SA, Jeraldo PR, Wilson JW, Khare R, Patel R. Metronidazole- and Carbapenem-Resistant Bacteroides thetaiotaomicron Isolated in Rochester, Minnesota, in 2014. Antimicrob Agents Chemother. 2015;59:4157–61. 10.1128/AAC.00677-15. PMC4468676 25941219

[44] Faye AS, Allin KH, Iversen AT, Agrawal M, Faith J, Colombel J-F, et al. Antibiotic use as a risk factor for inflammatory bowel disease across the ages: a population-based cohort study. Gut. 2023;72:663–70. 10.1136/gutjnl-2022-327845. PMC9998355 36623926

[45] Zhang Y, Xue G, Wang F, Zhang J, Xu L, Yu C. The impact of antibiotic exposure on antibiotic resistance gene dynamics in the gut microbiota of inflammatory bowel disease patients. Front Microbiol. 2024;15:1382332. 10.3389/fmicb.2024.1382332. PMC11061493 38694799

[46] Ravichandar JD, Rutherford E, Chow C-ET, Han A, Yamamoto ML, Narayan N, et al. Strain level and comprehensive microbiome analysis in inflammatory bowel disease via multi-technology meta-analysis identifies key bacterial influencers of disease. Front Microbiol. 2022;13:961020. 10.3389/fmicb.2022.961020. PMC9614153 36312950

[47] Fang X, Monk JM, Nurk S, Akseshina M, Zhu Q, Gemmell C, et al. Metagenomics-Based, Strain-Level Analysis of Escherichia coli From a Time-Series of Microbiome Samples From a Crohn’s Disease Patient. Front Microbiol. 2018;9:2559. 10.3389/fmicb.2018.02559. PMC6218438 30425690

[48] Clinical and Laboratory Standards Institute. Performance Standards for Antimicrobial Susceptibility Testing. 28th ed. CLSI supplement M100. Wayne, PA: Clinical and Laboratory Standards Institute; 2018.

[49] Miles AA, Misra SS, Irwin JO. The estimation of the bactericidal power of the blood. Epidemiol Infect. 1938;38:732–49. 10.1017/S002217240001158X. PMC2199673 20475467

[50] Bates D, Mächler M, Bolker B, Walker S. Fitting Linear Mixed-Effects Models Using lme4. J Stat Soft. 2015;67. 10.18637/jss.v067.i01.

[51] Kuznetsova A, Brockhoff PB, Christensen RHB. lmerTest Package: Tests in Linear Mixed Effects Models. J Stat Soft. 2017;82. 10.18637/jss.v082.i13.

[52] Lenth RV, Piaskowski J, emmeans M. 2017;2.0.3. 10.32614/CRAN.package.emmeans.

[53] Buchfink B, Xie C, Huson DH. Fast and sensitive protein alignment using DIAMOND. Nat Methods. 2015;12:59–60. 10.1038/nmeth.3176. 25402007

[54] Alcock BP, Huynh W, Chalil R, Smith KW, Raphenya AR, Wlodarski MA, et al. CARD 2023: expanded curation, support for machine learning, and resistome prediction at the Comprehensive Antibiotic Resistance Database. Nucleic Acids Res. 2023;51:D690–9. 10.1093/nar/gkac920. PMC9825576 36263822

[55] Savitzky Abraham, Golay MJE. Smoothing and Differentiation of Data by Simplified Least Squares Procedures. Anal Chem. 1964;36:1627–39. 10.1021/ac60214a047.

[56] Baranyi J, Roberts TA. A dynamic approach to predicting bacterial growth in food. Int J Food Microbiol. 1994;23:277–94. 10.1016/0168-1605(94)90157-0. 7873331

[57] Liu B, Garza DR, Gonze D, Krzynowek A, Simoens K, Bernaerts K, et al. Starvation responses impact interaction dynamics of human gut bacteria *Bacteroides thetaiotaomicron* and *Roseburia intestinalis*. ISME J. 2023;17:1940–52. 10.1038/s41396-023-01501-1. PMC10579405 37670028

[58] Garza DR, Liu B, Van De Velde C, Zhou X, Saha P, Gonze D, et al. Emergence of alternative states in a synthetic human gut microbial community. Nat Commun. 2025;17:326. 10.1038/s41467-025-67036-5. PMC12789478 41326405

[59] Ghotaslou R, Bannazadeh Baghi H, Alizadeh N, Yekani M, Arbabi S, Memar MY. Mechanisms of Bacteroides fragilis resistance to metronidazole. Infect Genet Evol. 2018;64:156–63. 10.1016/j.meegid.2018.06.020. 29936037

[60] Estabrook M, Muyldermans A, Sahm D, Pierard D, Stone G, Utt E. Epidemiology of Resistance Determinants Identified in Meropenem-Nonsusceptible *Enterobacterales* Collected as Part of a Global Surveillance Study, 2018 to 2019. Antimicrob Agents Chemother. 2023;67:e01406–22. 10.1128/aac.01406-22. PMC10190273 37074173

[61] Stentz R, Horn N, Cross K, Salt L, Brearley C, Livermore DM, et al. Cephalosporinases associated with outer membrane vesicles released by Bacteroides spp. protect gut pathogens and commensals against β-lactam antibiotics. J Antimicrob Chemother. 2015;70:701–9. 10.1093/jac/dku466. PMC4319488 25433011

[62] Parks DH, Imelfort M, Skennerton CT, Hugenholtz P, Tyson GW. CheckM: assessing the quality of microbial genomes recovered from isolates, single cells, and metagenomes. Genome Res. 2015;25:1043–55. 10.1101/gr.186072.114. PMC4484387 25977477

[63] Alosaimy S, Abdul-Mutakabbir JC, Kebriaei R, Jorgensen SCJ, Rybak MJ. Evaluation of Eravacycline: A Novel Fluorocycline. Pharmacotherapy. 2020;40:221–38. 10.1002/phar.2366. 31944332

[64] Falagas ME, Romanos LT, Kontogiannis DS, Filippou C, Karageorgopoulos DE. Resistance of Gram-Negative Bacteria to Eravacycline: A Systematic Review of Data from In Vitro Studies. Pathogens. 2025;14:1214. 10.3390/pathogens14121214. PMC12735846 41471170

[65] Haeili M, Aghajanzadeh M, Moghaddasi K, Omrani M, Ghodousi A, Cirillo DM. Emergence of transferable tigecycline and eravacycline resistance gene tet(X4) in Escherichia coli isolates from Iran. Sci Rep. 2025;15:16581. 10.1038/s41598-025-98206-6. PMC12075785 40360684

[66] Melville CM, Scott KP, Mercer DK, Flint HJ. Novel Tetracycline Resistance Gene, *tet* (32), in the *Clostridium* -Related Human Colonic Anaerobe K10 and Its Transmission In Vitro to the Rumen Anaerobe *Butyrivibrio fibrisolvens*. Antimicrob Agents Chemother. 2001;45:3246–9. 10.1128/AAC.45.11.3246-3249.2001. PMC90818 11600392

[67] Papp-Wallace KM, Endimiani A, Taracila MA, Bonomo RA, Carbapenems. Past, Present, and Future. Antimicrob Agents Chemother. 2011;55:4943–60. 10.1128/AAC.00296-11. PMC3195018 21859938

[68] Dingsdag SA, Hunter N. Metronidazole: an update on metabolism, structure–cytotoxicity and resistance mechanisms. J Antimicrob Chemother. 2018;73:265–79. 10.1093/jac/dkx351. 29077920

[69] Alauzet C, Lozniewski A, Marchandin H. Metronidazole resistance and nim genes in anaerobes: A review. Anaerobe. 2019;55:40–53. 10.1016/j.anaerobe.2018.10.004. 30316817

[70] Schapiro JM, Gupta R, Stefansson E, Fang FC, Limaye AP. Isolation of Metronidazole-Resistant *Bacteroides fragilis* Carrying the *nimA* Nitroreductase Gene from a Patient in Washington State. J Clin Microbiol. 2004;42:4127–9. 10.1128/JCM.42.9.4127-4129.2004. PMC516330 15364999

[71] Gin A, Dilay L, Karlowsky JA, Walkty A, Rubinstein E, Zhanel GG. Piperacillin–tazobactam: a β-lactam/β-lactamase inhibitor combination. Expert Rev Anti-infective Therapy. 2007;5:365–83. 10.1586/14787210.5.3.365. 17547502

[72] Rogers MB, Parker AC, Smith CJ. Cloning and characterization of the endogenous cephalosporinase gene, cepA, from Bacteroides fragilis reveals a new subgroup of Ambler class A beta-lactamases. Antimicrob Agents Chemother. 1993;37:2391–400. 10.1128/AAC.37.11.2391. PMC192397 8285623

[73] Campbell EA, Korzheva N, Mustaev A, Murakami K, Nair S, Goldfarb A, et al. Structural Mechanism for Rifampicin Inhibition of Bacterial RNA Polymerase. Cell. 2001;104:901–12. 10.1016/S0092-8674(01)00286-0. 11290327

[74] Goldstein BP. Resistance to rifampicin: a review. J Antibiot. 2014;67:625–30. 10.1038/ja.2014.107. 25118103

[75] Jin DJ, Gross CA. Mapping and sequencing of mutations in the Escherichia colirpoB gene that lead to rifampicin resistance. J Mol Biol. 1988;202:45–58. 10.1016/0022-2836(88)90517-7. 3050121

[76] Kitano K, Tomasz A. Triggering of autolytic cell wall degradation in Escherichia coli by beta-lactam antibiotics. Antimicrob Agents Chemother. 1979;16:838–48. 10.1128/AAC.16.6.838. PMC352963 93877

[77] Alvarez L, Hernandez SB, Torrens G, Weaver AI, Dörr T, Cava F. Control of bacterial cell wall autolysins by peptidoglycan crosslinking mode. Nat Commun. 2024;15:7937. 10.1038/s41467-024-52325-2. PMC11390936 39261529

[78] Yaffe E, Dethlefsen L, Patankar AV, Gui C, Holmes S, Relman DA. Brief antibiotic use drives human gut bacteria towards low-cost resistance. Nature. 2025;641:182–91. 10.1038/s41586-025-08781-x. PMC12168174 40269166

[79] Ledder O. Antibiotics in inflammatory bowel diseases: do we know what we’re doing? Transl Pediatr. 2019;8:42–55. 10.21037/tp.2018.11.02. PMC6382505 30881898

[80] Nitzan O. Role of antibiotics for treatment of inflammatory bowel disease. WJG. 2016;22:1078. 10.3748/wjg.v22.i3.1078. PMC4716021 26811648

[81] Crits-Christoph A, Hallowell HA, Koutouvalis K, Suez J. Good microbes, bad genes? The dissemination of antimicrobial resistance in the human microbiome. Gut Microbes. 2022;14:2055944. 10.1080/19490976.2022.2055944. PMC8959533 35332832

[82] Lee K, Raguideau S, Sirén K, Asnicar F, Cumbo F, Hildebrand F, et al. Population-level impacts of antibiotic usage on the human gut microbiome. Nat Commun. 2023;14:1191. 10.1038/s41467-023-36633-7. PMC9981903 36864029

[83] Sóki J, Wybo I, Baaity Z, Stefán G, Jeverica S, Ulger N, et al. Detection of the antibiotic resistance genes content of intestinal Bacteroides, Parabacteroides and Phocaeicola isolates from healthy and carbapenem-treated patients from European countries. BMC Microbiol. 2024;24:202. 10.1186/s12866-024-03354-w. PMC11162026 38851699

[84] Diebold PJ, Rhee MW, Shi Q, Trung NV, Umrani F, Ahmed S, et al. Clinically relevant antibiotic resistance genes are linked to a limited set of taxa within gut microbiome worldwide. Nat Commun. 2023;14:7366. 10.1038/s41467-023-42998-6. PMC10645880 37963868

[85] Fishbein SRS, Mahmud B, Dantas G. Antibiotic perturbations to the gut microbiome. Nat Rev Microbiol. 2023;21:772–88. 10.1038/s41579-023-00933-y. PMC12087466 37491458

[86] Diamantis S, Retur N, Bertrand B, Lieutier-Colas F, Carenco P, Mondain V, et al. The Production of Antibiotics Must Be Reoriented: Repositioning Old Narrow-Spectrum Antibiotics, Developing New Microbiome-Sparing Antibiotics. Antibiotics. 2022;11:924. 10.3390/antibiotics11070924. PMC9311687 35884178

[87] Rea MC, Dobson A, O’Sullivan O, Crispie F, Fouhy F, Cotter PD, et al. Effect of broad- and narrow-spectrum antimicrobials on *Clostridium difficile* and microbial diversity in a model of the distal colon. Proc Natl Acad Sci USA. 2011;108 supplement1:4639–44. 10.1073/pnas.1001224107. PMC3063588 20616009

[88] Qian X, Yanagi K, Kane AV, Alden N, Lei M, Snydman DR, et al. Ridinilazole, a narrow spectrum antibiotic for treatment of *Clostridioides difficile* infection, enhances preservation of microbiota-dependent bile acids. Am J Physiology-Gastrointestinal Liver Physiol. 2020;319:G227–37. 10.1152/ajpgi.00046.2020. PMC7500266 32597706

[89] Catacutan DB, Tran V, Arnold A, Alexander J, Corso G, Yousefi Y, et al. Discovery and artificial intelligence-guided mechanistic elucidation of a narrow-spectrum antibiotic. Nat Microbiol. 2025;10:2808–22. 10.1038/s41564-025-02142-0. 41044363

[90] Greulich P, Scott M, Evans MR, Allen RJ. Growth-dependent bacterial susceptibility to ribosome‐targeting antibiotics. Mol Syst Biol. 2015;11:796. 10.15252/msb.20145949. PMC4380930 26146675

[91] Hromada S, Venturelli OS. Gut microbiota interspecies interactions shape the response of Clostridioides difficile to clinically relevant antibiotics. PLoS Biol. 2023;21:e3002100. 10.1371/journal.pbio.3002100. PMC10174544 37167201

[92] Garcia-Santamarina S, Kuhn M, Devendran S, Maier L, Driessen M, Mateus A, et al. Emergence of community behaviors in the gut microbiota upon drug treatment. Cell. 2024;187:6346–e635720. 10.1016/j.cell.2024.08.037. 39321801

