# Supplementary material for: Effects of common antibiotics on representative gut bacterial strains associated with inflammatory bowel disease
Source: BMC Microbiol. 2026 Aug 6;26:965. doi: 10.1186/s12866-026-05459-w (PMC13737694; doi:10.1186/s12866-026-05459-w)
Supplement: Supplementary file 1 [file 12866_2026_5459_MOESM1_ESM.docx]

**SUPPLEMENTARY MATERIAL**

1. **Supplementary Figures**

**Supplementary Figure S1**. Experimental design and growth curve analysis workflow**.** (A) Schematic of the strains used in this study and their role in the human gut microbiome: *Bacteroides thetaiotaomicron*, *Blautia hydrogenotrophica*, *Roseburia intestinalis*, *Bacteroides fragilis* and *Escherichia coli* LF82; (B) Plate layout used for the two-fold broth dilution of the antibiotics, including negative and positive control wells. Each row is assigned to one antibiotic (or a combination such as piperacillin/tazobactam); (C) Schematic overview of the experimental workflow to determine the antibiotic susceptibility of the monocultures. The plates were inoculated and incubated anaerobically in a plate reader for 48 hours at 37 °C and OD was recorded over time. The OD curves were fit with a Monod-type ODE model (See Methods). Created in BioRender.

**Supplementary Figure S2.** Goodness of fit of the Monod-type growth model across antibiotic concentrations. Root means square error (RMSE) between experimentally measured OD time series and model-predicted growth curves is shown as a function of antibiotic concentration for: *B. fragilis* (A), *E. coli* LF82 (B), *B. thetaiotaomicron* (C) and *B. hydrogenotrophica* (D).

**Supplementary Figure S3.** Relationships between growth curve-derived slopes and model-inferred growth and death rates. Plots showing the association between empirical growth curve characteristics and parameters inferred from the Monod-type ODE model for each strain-antibiotic combination: *B. fragilis* (A), *E. coli* LF82 (B), *B. thetaiotaomicron* (C) and *B. hydrogenotrophica* (D). Panels show (a) maximum slope of the OD curve is compared with the estimated growth rate and (b) the absolute value of the minimum slope is compared with the estimated death rate. Spearman correlation coefficients ($\rho_{X,Y}$) are reported above each panel to quantify associations between slope-based metrics and model-derived parameters.

**Supplementary Figure S4.** High concentration antibiotic effects on gut bacterial growth dynamics. OD growth curves of five bacterial strains measured over time in the presence of five different concentrations of antibiotics. Rows correspond to antibiotics: Eravacycline (A), Meropenem (B), Metronidazole (C), Piperacillin/Tazobactam (D) and Rifampin (E). Columns correspond to bacterial members: *B. fragilis* (BF), *E. coli* LF82 (EC), *B. thetaiotaomicron* (BT). BF and BT were tested for all antibiotics, while EC was tested for only eravacycline, piperacillin/tazobactam and rifampin.

**Supplementary Figure S5.** Maximum slopes decay exponentially with antibiotic concentration. (A) Exponential decay fitted to maximum slopes of growth curves in low and high antibiotic concentration ranges for *E. coli* LF82. (B) Exponential decay curves were derived only based on maximum slopes at low concentrations and extrapolated to high concentrations for *E. coli* LF82. Fitting was carried out with non-linear least squares implemented using the R function *nls*.

**Supplementary Figure S6.** Comparison of growth curve characteristics with model parameters across antibiotic concentrations. Growth curve characteristics derived from the OD time series are shown as a function of antibiotic concentration for: *B. fragilis* (A), *E. coli* LF82 (B), *B. thetaiotaomicron* (C) and *B. hydrogenotrophica* (D). Panels show maximum OD (a), final OD at 48h (b), maximum slope (c) and minimum slope (d).

**Supplementary Figure S7.** Viable cell counts (CFU/mL) of *B. fragilis* (A) and *E. coli* LF82 (B) following exposure to meropenem. Cultures were grown without antibiotic (control) or with meropenem at 0.2 µg/mL (low) or 2.0 µg/mL (high), and sampled after 8 h and 24 h. Violin plots show the distribution of CFU measurements on a logarithmic y-axis, with points representing replicate observations. Statistical analyses were performed using linear mixed-effects models to assess the effects of meropenem concentration, timepoint, which treated the replicates as random effects (see Methods).

**Supplementary Figure S8.** Growth dynamics of *E. coli* LF82 (A) and *B. fragilis* (B) under meropenem treatment. Optical density (OD) was monitored over time for *E. coli* LF82 and *B. fragilis* grown without meropenem (0 µg/mL), with low meropenem concentration (0.2 µg/mL), or with high meropenem concentration (2 µg/mL). Lines show the mean OD over time, and shaded regions indicate variability between replicates.

**Supplementary Figure S9.** Minimum inhibitory concentrations (MICs) of five antibiotics against five gut bacterial strains. Each symbol plotted represents one strain–antibiotic combination, with MIC values plotted on a logarithmic y-axis. Filled symbols indicate that complete growth inhibition was observed within the tested concentration range (endpoint reached). Open symbols indicate that growth was not fully inhibited at the highest concentration tested; the plotted value is therefore a lower bound on the true MIC. Here Pip/Taz represents piperacillin/tazobactam.

2. **Supplementary Tables**

**Supplementary Table S1.** Summary of 16S rRNA gene Sanger sequencing BLAST results for monoculture purity verification.

| **Sample label** | **Expected Strain** | **Sampling point** | **Top BLAST Hit** | **Percent Identity** | **Query Coverage** | **Accession** |
| --- | --- | --- | --- | --- | --- | --- |
| PS_Bf_250825 | *B. fragilis* NCTC 9343 | Preculture | *B. fragilis* NCTC 9343 | 98.94% | 99% | [NR_074784.2](https://www.ncbi.nlm.nih.gov/nucleotide/NR_074784.2?report=genbank&log$=nucltop&blast_rank=1&RID=23WGR25T014) |
| PS_Bf_250825_MT_C2 | *B. fragilis* NCTC 9343 | Post experiment | *B. fragilis* NCTC 9343 | 98.94% | 99% | [NR_074784.2](https://www.ncbi.nlm.nih.gov/nucleotide/NR_074784.2?report=genbank&log$=nucltop&blast_rank=1&RID=23WGR25T014) |
| PS_Bf_250825_E_C2 | *B. fragilis* NCTC 9343 | Post experiment | *B. fragilis* NCTC 9343 | 98.92% | 99% | [NR_074784.2](https://www.ncbi.nlm.nih.gov/nucleotide/NR_074784.2?report=genbank&log$=nucltop&blast_rank=1&RID=23WHV2M9016) |
| PS_Bf_250825_R_C2 | *B. fragilis* NCTC 9343 | Post experiment | *B. fragilis* NCTC 9343 | 99.13% | 99% | [NR_074784.2](https://www.ncbi.nlm.nih.gov/nucleotide/NR_074784.2?report=genbank&log$=nucltop&blast_rank=1&RID=23WHV2M9016) |
| PS_Bf_250825_PC_C8 | *B. fragilis* NCTC 9343 | Post experiment | *B. fragilis* NCTC 9343 | 99.23% | 99% | [NR_074784.2](https://www.ncbi.nlm.nih.gov/nucleotide/NR_074784.2?report=genbank&log$=nucltop&blast_rank=1&RID=23WHV2M9016) |
| PS_Bf_250825_M_C2 | *B. fragilis* NCTC 9343 | Post experiment | *B. fragilis* NCTC 9343 | 99.3% | 99% | [NR_074784.2](https://www.ncbi.nlm.nih.gov/nucleotide/NR_074784.2?report=genbank&log$=nucltop&blast_rank=1&RID=23WHV2M9016) |
| PS_Bf_250825_PT_C2 | *B. fragilis* NCTC 9343 | Post experiment | *B. fragilis* NCTC 9343 | 99.39% | 99% | [NR_074784.2](https://www.ncbi.nlm.nih.gov/nucleotide/NR_074784.2?report=genbank&log$=nucltop&blast_rank=1&RID=23WHV2M9016) |
| PS_Ec_250825 | *E. coli* LF82*^a^* | Preculture | *E. fergusonii* | 98.95% | 99% | [NR_074902.1](https://www.ncbi.nlm.nih.gov/nucleotide/NR_074902.1?report=genbank&log$=nucltop&blast_rank=1&RID=23X1S8KJ014) |
| PS_Ec_250825_MT_C9 | *E. coli* LF82*^a^* | Post experiment | *E. fergusonii* | 97.58% | 99% | [NR_074902.1](https://www.ncbi.nlm.nih.gov/nucleotide/NR_074902.1?report=genbank&log$=nucltop&blast_rank=1&RID=23X1S8KJ014) |
| PS_Ec_250825_E_C9 | *E. coli* LF82*^a^* | Post experiment | *E. fergusonii* | 98.20% | 99% | [NR_074902.1](https://www.ncbi.nlm.nih.gov/nucleotide/NR_074902.1?report=genbank&log$=nucltop&blast_rank=1&RID=23X1S8KJ014) |
| PS_Ec_250825_R_C9 | *E. coli* LF82*^a^* | Post experiment | *E. fergusonii* | 98.54% | 99% | [NR_074902.1](https://www.ncbi.nlm.nih.gov/nucleotide/NR_074902.1?report=genbank&log$=nucltop&blast_rank=1&RID=23X1S8KJ014) |
| PS_Ec_250825_PC_C9 | *E. coli* LF82*^a^* | Post experiment | *E. fergusonii* | 98.32% | 99% | [NR_074902.1](https://www.ncbi.nlm.nih.gov/nucleotide/NR_074902.1?report=genbank&log$=nucltop&blast_rank=1&RID=23X1S8KJ014) |
| PS_Ec_250825_M_C9 | *E. coli* LF82*^a^* | Post experiment | *E. fergusonii* | 98.89% | 99% | [NR_074902.1](https://www.ncbi.nlm.nih.gov/nucleotide/NR_074902.1?report=genbank&log$=nucltop&blast_rank=1&RID=23X1S8KJ014) |
| PS_Ec_250825_PT_C9 | *E. coli* LF82*^a^* | Post experiment | *E. fergusonii* | 99.45% | 99% | [NR_074902.1](https://www.ncbi.nlm.nih.gov/nucleotide/NR_074902.1?report=genbank&log$=nucltop&blast_rank=1&RID=23X1S8KJ014) |
| PS_Bt_250825 | *B. thetaiotaomicron* VPI 5482 | Preculture | *B. thetaiotaomicron* VPI 5482 | 98.65% | 99% | [NR_074277.1](https://www.ncbi.nlm.nih.gov/nucleotide/NR_074277.1?report=genbank&log$=nucltop&blast_rank=2&RID=2427XB6E016) |
| PS_Bt_250825_MT_C2 | *B. thetaiotaomicron* VPI 5482 | Post experiment | *B. thetaiotaomicron* VPI 5482 | 97.28% | 99% | [NR_074277.1](https://www.ncbi.nlm.nih.gov/nucleotide/NR_074277.1?report=genbank&log$=nucltop&blast_rank=2&RID=2427XB6E016) |
| PS_Bt_250825_E_C2 | *B. thetaiotaomicron* VPI 5482 | Post experiment | *B. thetaiotaomicron* VPI 5482 | 98.46% | 98% | [NR_074277.1](https://www.ncbi.nlm.nih.gov/nucleotide/NR_074277.1?report=genbank&log$=nucltop&blast_rank=2&RID=2427XB6E016) |
| PS_Bt_250825_R_C2 | *B. thetaiotaomicron* VPI 5482 | Post experiment | *B. thetaiotaomicron* VPI 5482 | 98.67% | 99% | [NR_074277.1](https://www.ncbi.nlm.nih.gov/nucleotide/NR_074277.1?report=genbank&log$=nucltop&blast_rank=2&RID=2427XB6E016) |
| PS_Bt_250825_PC_C8 | *B. thetaiotaomicron* VPI 5482 | Post experiment | *B. thetaiotaomicron* VPI 5482 | 97.47% | 99% | [NR_074277.1](https://www.ncbi.nlm.nih.gov/nucleotide/NR_074277.1?report=genbank&log$=nucltop&blast_rank=2&RID=2427XB6E016) |
| PS_Bt_250825_M_C2 | *B. thetaiotaomicron* VPI 5482 | Post experiment | *B. thetaiotaomicron* VPI 5482 | 97.88% | 99% | [NR_074277.1](https://www.ncbi.nlm.nih.gov/nucleotide/NR_074277.1?report=genbank&log$=nucltop&blast_rank=2&RID=2427XB6E016) |
| PS_Bt_250825_PT_C2 | *B. thetaiotaomicron* VPI 5482 | Post experiment | *B. thetaiotaomicron* VPI 5482 | 97.39% | 99% | [NR_074277.1](https://www.ncbi.nlm.nih.gov/nucleotide/NR_074277.1?report=genbank&log$=nucltop&blast_rank=2&RID=2427XB6E016) |
| PS_Bh_060925 | *B. hydrogenotrophica* DSM 10507 | Preculture | *B. hydrogenotrophica* | 98.81% | 98% | [NR_026314.1](https://www.ncbi.nlm.nih.gov/nucleotide/NR_026314.1?report=genbank&log$=nucltop&blast_rank=1&RID=244HNBN1014) |
| PS_Bh_060925_MT_B2 | *B. hydrogenotrophica* DSM 10507 | Post experiment | *B. hydrogenotrophica* | 99.06% | 99% | [NR_026314.1](https://www.ncbi.nlm.nih.gov/nucleotide/NR_026314.1?report=genbank&log$=nucltop&blast_rank=1&RID=244HNBN1014) |
| PS_Bh_060925_E_C7 | *B. hydrogenotrophica* DSM 10507 | Post experiment | *B. hydrogenotrophica* | 99.49% | 99% | [NR_026314.1](https://www.ncbi.nlm.nih.gov/nucleotide/NR_026314.1?report=genbank&log$=nucltop&blast_rank=1&RID=244HNBN1014) |
| PS_Bh_060925_R_D7 | *B. hydrogenotrophica* DSM 10507 | Post experiment | *B. hydrogenotrophica* | 99.10% | 99% | [NR_026314.1](https://www.ncbi.nlm.nih.gov/nucleotide/NR_026314.1?report=genbank&log$=nucltop&blast_rank=1&RID=244HNBN1014) |
| PS_Bh_060925_PC_E7 | *B. hydrogenotrophica* DSM 10507 | Post experiment | *B. hydrogenotrophica* | 99.42% | 99% | [NR_026314.1](https://www.ncbi.nlm.nih.gov/nucleotide/NR_026314.1?report=genbank&log$=nucltop&blast_rank=1&RID=244HNBN1014) |
| PS_Bh_060925_PT_G7 | *B. hydrogenotrophica* DSM 10507 | Post experiment | *B. hydrogenotrophica* | 98.18% | 99% | [NR_026314.1](https://www.ncbi.nlm.nih.gov/nucleotide/NR_026314.1?report=genbank&log$=nucltop&blast_rank=1&RID=244HNBN1014) |
| PS_Bh_060925_M_F7 | *B. hydrogenotrophica* DSM 10507 | Post experiment | *B. hydrogenotrophica* | 98.82% | 98% | [NR_026314.1](https://www.ncbi.nlm.nih.gov/nucleotide/NR_026314.1?report=genbank&log$=nucltop&blast_rank=1&RID=244HNBN1014) |
| PS_Ri_040925 | *R. intestinalis* L1-82 | Preculture | *R. intestinalis* L1-82 | 98.84% | 99% | [NR_027557.1](https://www.ncbi.nlm.nih.gov/nucleotide/NR_027557.1?report=genbank&log$=nucltop&blast_rank=1&RID=23WNEF4E014) |
| PS_Ri_040925_PT_G7 | *R. intestinalis* L1-82 | Post experiment | *R. intestinalis* L1-82 | 99.10% | 99% | [NR_027557.1](https://www.ncbi.nlm.nih.gov/nucleotide/NR_027557.1?report=genbank&log$=nucltop&blast_rank=1&RID=23WNEF4E014) |
| PS_Ri_040925_PC_E7 | *R. intestinalis* L1-82 | Post experiment | *R. intestinalis* L1-82 | 98.94% | 99% | [NR_027557.1](https://www.ncbi.nlm.nih.gov/nucleotide/NR_027557.1?report=genbank&log$=nucltop&blast_rank=1&RID=23WNEF4E014) |
| PS_Ri_040925_MT_B8 | *R. intestinalis* L1-82 | Post experiment | *R. intestinalis* L1-82 | 97.91% | 99% | [NR_027557.1](https://www.ncbi.nlm.nih.gov/nucleotide/NR_027557.1?report=genbank&log$=nucltop&blast_rank=1&RID=23WNEF4E014) |
| PS_Ri_040925_E_C7 | *R. intestinalis* L1-82 | Post experiment | *R. intestinalis* L1-82 | 97.83% | 99% | [NR_027557.1](https://www.ncbi.nlm.nih.gov/nucleotide/NR_027557.1?report=genbank&log$=nucltop&blast_rank=1&RID=23WNEF4E014) |
| PS_Ri_040925_R_D7 | *R. intestinalis* L1-82 | Post experiment | *R. intestinalis* L1-82 | 98.09% | 99% | [NR_027557.1](https://www.ncbi.nlm.nih.gov/nucleotide/NR_027557.1?report=genbank&log$=nucltop&blast_rank=1&RID=23WNEF4E014) |
| PS_Ri_040925_M_F7 | *R. intestinalis* L1-82 | Post experiment | *R. intestinalis* L1-82 | 98.63% | 99% | [NR_027557.1](https://www.ncbi.nlm.nih.gov/nucleotide/NR_027557.1?report=genbank&log$=nucltop&blast_rank=1&RID=23WNEF4E014) |

*^a^* For *E. coli* LF82, 16S rRNA gene sequencing identified *Escherichia fergusonii* as the top BLAST hit. This is expected because the 16S rRNA gene does not provide sufficient resolution to distinguish closely related *Escherichia* species and strains. Strain identity was therefore independently confirmed by whole-genome sequencing (Supplementary Table S2).

**Supplementary table S2. Whole-genome sequence confirmation of *E. coli* LF82.**

| **Strain** | **Reference genome** | **Genome coverage (%)** | **ANI (%)** |
| --- | --- | --- | --- |
| *Escherichia coli* LF82 | BVBRC Genome  [591946.4](https://www.bv-brc.org/view/Genome/591946.4) | 99.1758 | 99.9951 |

**Note:** Whole-genome sequence analysis was performed independently of the plate-reader experiments. Reads were pre-processed using fastp, assembled using SPAdes, aligned to the *E. coli* LF82 reference genome (BVBRC Genome 591946.4) using minimap2 (v. 2.28-r1209), and genome coverage was calculated using samtools (samtools v. 1.9). Average nucleotide identity (ANI) was calculated using fastANI ( v. 1.34).
